# High-precision ^81^Kr dating overcomes radiocarbon limits in Late Pleistocene groundwater

**DOI:** 10.1126/sciadv.aef6864

**Published:** 2026-11-13

**Authors:** Hao Li, Guo-Min Yang, Xilin Bao, Wei Jiang, Zheng-Tian Lu, Huifeng Yang, Mengjie Zhang, Lei Zhao

**Affiliations:** ^1^ Hefei National Research Center for Physical Sciences at the Microscale and School of Physical Sciences, University of Science and Technology of China, Hefei 230026, China.; ^2^ Hefei National Laboratory, University of Science and Technology of China, Hefei 230088, China.; ^3^ Institute of Hydrogeology and Environmental Geology, Chinese Academy of Geological Sciences, Shijiazhuang 050061, China.

## Abstract

Groundwater preserves continental hydroclimate signals, but its use as a Late Pleistocene archive is limited by uncertainties in radiocarbon (^14^C) dating caused by carbon geochemical processes. The noble gas isotope ^81^Kr has simpler transport behavior, yet its effective dating limit was traditionally ∼50 thousand years (ka) because of analytical constraints. Here, we extended the ^81^Kr dating lower limit to ∼10 ka using high-precision atom trap trace analysis, thus covering the entire Late Pleistocene. We conducted a direct ^14^C-^81^Kr comparison in the North China Plain. Within the 10- to 30-ka range, ^81^Kr independently validates ^14^C ages; beyond this range, it provides reliable ages for older groundwater. The dual-tracer approach reduces model uncertainty in ^14^C correction and identifies mixing-affected samples. After screening, groundwater δ^18^O records closely match polar ice-core climate archives. High-precision ^81^Kr dating, combined with ^14^C, offers a robust age framework for reconstructing Late Pleistocene hydroclimate.

## INTRODUCTION

Reconstructing past climate requires archives that record environmental change across space and time. Ice cores, marine sediments, and speleothems provide such records, but they are often restricted to specific depositional settings. Groundwater, by contrast, is globally distributed in continental aquifers and integrates environmental signals over large scales, making it a valuable, yet underused, complementary archive of geological and climatic history (*1*–*4*). A prerequisite for exploiting this archive is accurate age determination.

The most commonly used method for dating ancient groundwater is radiocarbon [^14^C; half-life = 5.73 thousand years(ka)] (*5*). However, because of the complex geochemical behavior of carbon isotopes in groundwater, this method carries a major caveat. The initial ^14^C activity of dissolved inorganic carbon (DIC) is jointly controlled by soil carbon dioxide (CO_2_) input, carbonate dissolution, and open-system versus closed-system exchange. Therefore, geochemical correction models are essential to obtain reliable ^14^C ages. However, in practice, because of uncertainties in various initial parameters, the selection of a model is subject to subjectivity and arbitrariness. Moreover, when sample ages approach 30 to 40 ka, the low abundance of ^14^C amplifies both the age discrepancies among different correction models and the influence of modern carbon contamination, further increasing the uncertainty of ^14^C ages (*6*–*10*). Last, for groundwater older than 40 ka, meaningful ages cannot be obtained because this exceeds the dating range of ^14^C. These limitations restrict the application of ^14^C in ancient groundwater studies.

In contrast, the radioactive isotope ^81^Kr, with a half-life of 229 ± 11 ka (*11*, *12*), extends the dating range of groundwater to beyond 1 million years. As a noble gas isotope, ^81^Kr is homogeneously distributed in the atmosphere and exhibits relatively simple geochemical behavior in groundwater systems (*11*), making it an ideal tracer for investigating large-scale aquifer evolution (*13*–*18*). The development of atom trap trace analysis (ATTA) has enabled the measurement of ^81^Kr in environmental samples (see fig. S1) (*19*–*21*). However, previous analytical precision was ∼5% of modern abundance, corresponding to a lower dating limit of around 50 ka (see Materials and Methods), which precluded direct comparison with the ^14^C method.

In this study, we developed a high-precision ATTA method for ^81^Kr, improving the measurement precision from 5 to 1% (see Materials and Methods), thereby extending the lower dating limit to ∼10 ka. This advancement allows the ^81^Kr dating method to cover the entire Late Pleistocene (129 to 11.7 ka calibrated years before present), a critical period in geological history. We applied this technique to groundwater in the North China Plain, achieving a direct intercomparison between ^14^C and ^81^Kr ages. By combining ^81^Kr and ^14^C, we can obtain reliable ages for ancient groundwater and, within this robust chronological framework, compare its δ^18^O record with polar ice core climate archives, demonstrating the potential of ancient groundwater as a paleoclimate archive.

## RESULTS

### Sampling location and methods

The comparative study of ^14^C and ^81^Kr dating of groundwater was carried out on samples collected from the North China Plain. The regional Quaternary aquifer system is composed of alluvial-fan and lacustrine deposits and can be subdivided into four aquifer units on the basis of lithology, depositional age, aquifer-aquitard distribution, and hydrodynamic conditions (*22*). Owing to the extensive utilization of groundwater in this area, reliable age information is crucial for understanding recharge history and residence time and for supporting groundwater modeling efforts (*23*, *24*). Figure 1 shows the study area, sampling locations, and a cross-sectional profile of the region (*25*).

**Fig. 1.**
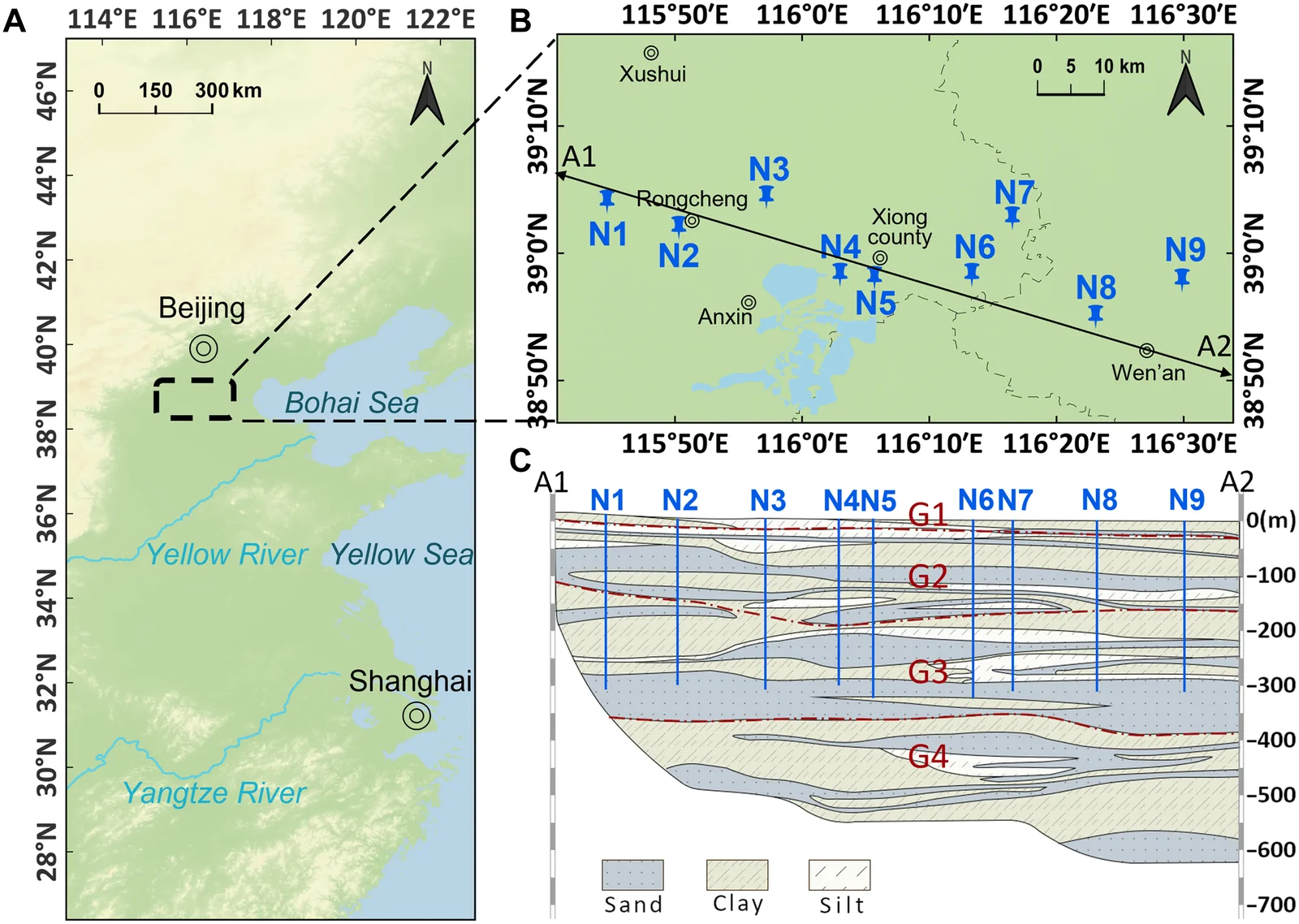
Sampling area in the North China Plain. (**A**) Map of the study area in the North China Plain. The dashed rectangle indicates the sampling area. (**B**) Map of the sampling area. The A1-A2 line marks the location of the following hydrogeological profile. (**C**) Hydrogeological profile along the A1-A2 line. Blue vertical lines indicate well depths. All groundwater samples are extracted from the G3 aquifer. Panel (C) was adapted with permission from the image on page 50 of *Atlas of Groundwater Sustainable Utilization in the North China Plain* (*25*).

We collected groundwater samples from nine wells within the G3 aquifer (Fig. 1C). ^14^C abundances of DIC in the groundwater samples were measured with accelerator mass spectrometry and δ^13^C and δ^18^O values with mass spectrometry, both by Beta Analytic Inc. For ^81^Kr, dissolved gas samples were collected on-site from groundwater using a degassing membrane unit (*26*). The extracted gases were transported to the University of Science and Technology of China laboratory, where the samples were purified to yield krypton (*27*), and ^81^Kr/Kr ratios were measured using the high-precision ATTA system. This system features a high ^81^Kr counting rate at a level of 6000 atoms/hour for a modern sample. The total ^81^Kr/Kr measurement uncertainty, which combines both statistical and systematic components, is at 1% of the modern level. Correspondingly, the age uncertainty for young samples is reduced to ±3 ka, extending the lower limit of ^81^Kr dating to 10 ka. Meanwhile, the ^85^Kr/Kr ratios in the samples were measured in parallel to assess atmospheric contamination. Details about the instrument, the well information, and raw isotope data are presented in the Supplementary Materials (see figs. S1 to S4 and tables S2 and S3).

### Comparison between the ^81^Kr and ^14^C data and ^14^C correction model selections

We first compared the measured abundances of ^81^Kr and ^14^C. As shown in Fig. 2A, the horizontal axis represents the measured ^81^Kr abundance ($R_{81}$) in pMKr (percent Modern Kr), and the vertical axis represents the measured ^14^C abundance ($R_{14}$) in pMC (percent Modern Carbon). The red curve in the figure represents the theoretical relationship when both isotopic ages are equal, given by the equation

Age=−t1/2(C14)ln 2·ln R14=−t1/2(Kr81)ln 2·ln R81 (1)

**Fig. 2.**
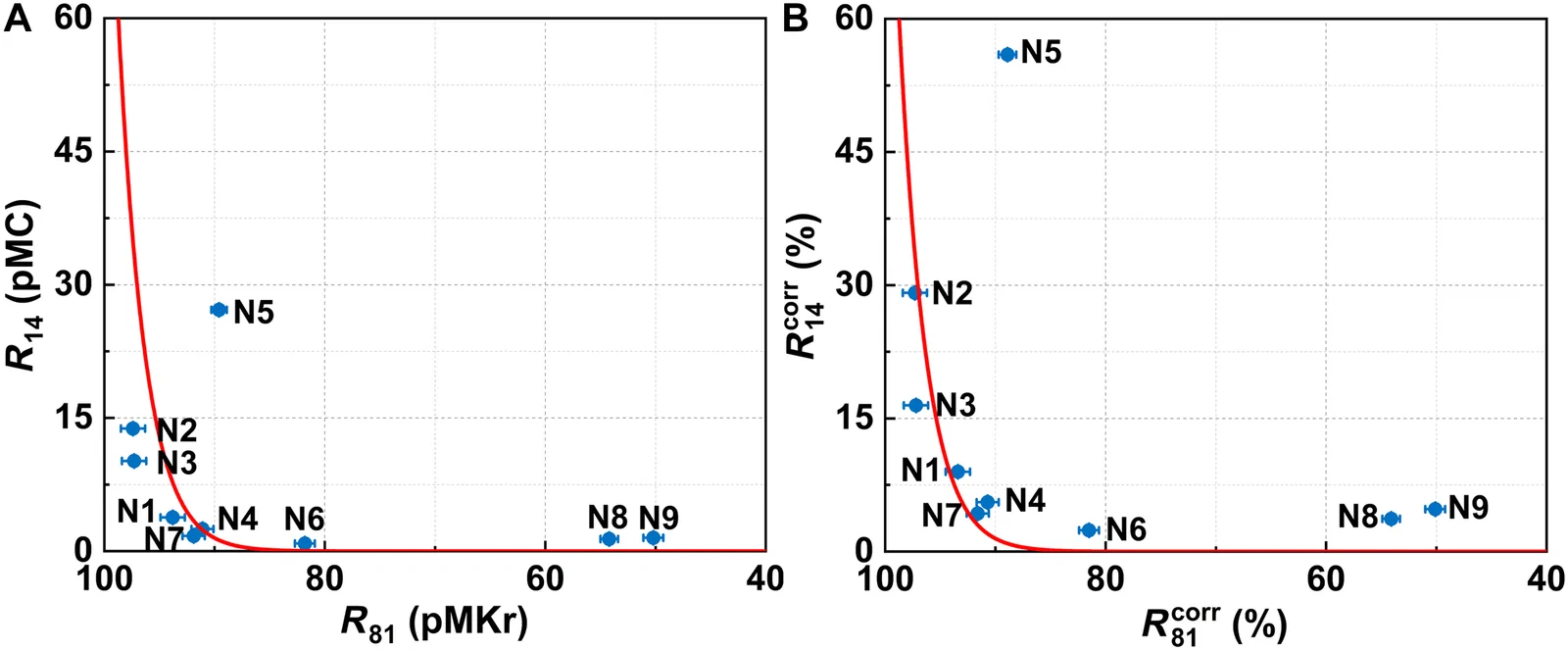
Comparison of ^14^C and ^81^Kr results. (**A**) Measured ^81^Kr and ^14^C. (**B**) Corrected ^81^Kr and ^14^C. The red curves in both panels represent the theoretical relationship when the two isotopic ages are equal.

In Fig. 2A, the data fall into two distinct groups. The first group (N1 to N4 and N7) lies close to the theoretical curve, suggesting that groundwater transport approximates ideal piston flow. The other group (N5, N6, N8, and N9) deviates substantially from the theoretical curve. Among these, N6, N8, and N9 have ^14^C content below 2 pMC and are susceptible to modern contamination during sampling and measurement, resulting in systematic deviations of similar magnitude from the theoretical curve. For N5, this deviation more likely reflects the influence of groundwater mixing or dispersion.

To enable a more quantitative comparison with the ^81^Kr data, we first performed corrections for all ^14^C data. This is because the ^14^C content in groundwater is controlled not only by radioactive decay but also by the initial composition of DIC and its subsequent geochemical evolution. These processes typically result in an initial ^14^C content of less than 100 pMC, which needs to be corrected. Previous studies have considered various geochemical models for the evolution of ^14^C in groundwater using measured δ^13^C values to correct the initial ^14^C content. These models rely on the major carbon species of DIC or the mass balance of carbon isotopes (^14^C and ^13^C). Consequently, the accuracy of ^14^C age estimation depends not only on the correct application of the model but also on the estimation of the ^14^C and δ^13^C values of soil CO_2_ and carbonate minerals. Correction models based on different geochemical processes yield different initial ^14^C content, and the choice of model substantially affects the calculated ^14^C age (see tables S7 and S8). Therefore, selecting an appropriate model for ^14^C corrections remains a challenging task.

To address this issue, Han and Plummer (*28*) developed a method on the basis of a ^14^C versus δ^13^C diagram to select an appropriate ^14^C correction model (see Materials and Methods). This diagram assigns different geochemical processes to distinct regions, including carbonate dissolution, gas exchange, and methanogenesis, and can be used to identify the dominant geochemical processes controlling ^14^C and δ^13^C evolution in groundwater, guiding the selection of the corresponding initial ^14^C correction model. However, some model parameters (e.g., the δ^13^C value of soil CO_2_) have substantial uncertainties, leading to systematic biases in the calculated ^14^C ages. This effect has not been accounted for in the uncertainty estimates of ^14^C ages in many studies. Thus, even with the Han and Plummer method, considerable uncertainty remains in the selection of an appropriate ^14^C correction model for groundwater. The ^81^Kr data in this study provide an opportunity to test and compare different ^14^C correction models. Owing to the ideal properties of ^81^Kr, its abundance can be directly converted into reliable groundwater ages. This allows us to evaluate various ^14^C correction models and identify the most appropriate one.

Figure 3 presents the Han and Plummer diagram along with our data. These models correspond to different geochemical processes; for example, the Eichinger model reflects isotopic exchange between groundwater and solid carbonates under closed-system conditions, whereas the Mook model applies to open-system conditions where groundwater continues to exchange with soil CO_2_. Most of our samples fall within the region applicable to the Eichinger model, with only N3 falling within the Mook model region. On the basis of the position of each sample in the Han-Plummer diagram, we selected the corresponding model to calculate the initial ^14^C content, then computed $R_{14}^{\text{corr}}$, and compared it with the ^81^Kr abundance data (Fig. 2B and Table 1; see tables S4 and S6 for details of the correction calculations). Figure 2B shows the relationship between ^81^Kr abundance and the corrected ^14^C content, where the theoretical curve follows the same form as Eq. 1. The horizontal axis represents the corrected ^81^Kr abundance ($R_{81}^{\text{corr}}$), defined as $R_{81}^{\text{corr}}=R_{81}/R_{81}^{\text{init}}$, where $R_{81}^{\text{init}}$ is the initial ^81^Kr abundance. $R_{81}^{\text{init}}$ has been corrected for the atmospheric input function of ^81^Kr (see fig. S5) (*19*). Because the atmospheric input history of ^81^Kr is relatively stable, the correction to ^81^Kr is less than 0.5%. The vertical axis represents the corrected ^14^C content ($R_{14}^{\text{corr}}$), defined as $R_{14}^{\text{corr}}=R_{14}/R_{14}^{\text{init}}$, where $R_{14}^{\text{init}}$ is the initial ^14^C content calculated using NETPATH software (see Materials and Methods and table S7). As shown in fig. S2B, for the first group of samples (N1 to N4 and N7), all except N4 show better agreement with the theoretical curve after correction. For the second group (N5, N6, N8, and N9), the deviation from the theoretical curve becomes even larger after correction, confirming our previous inference that these samples are near the ^14^C dating limit or have been affected by mixing.

**Fig. 3.**
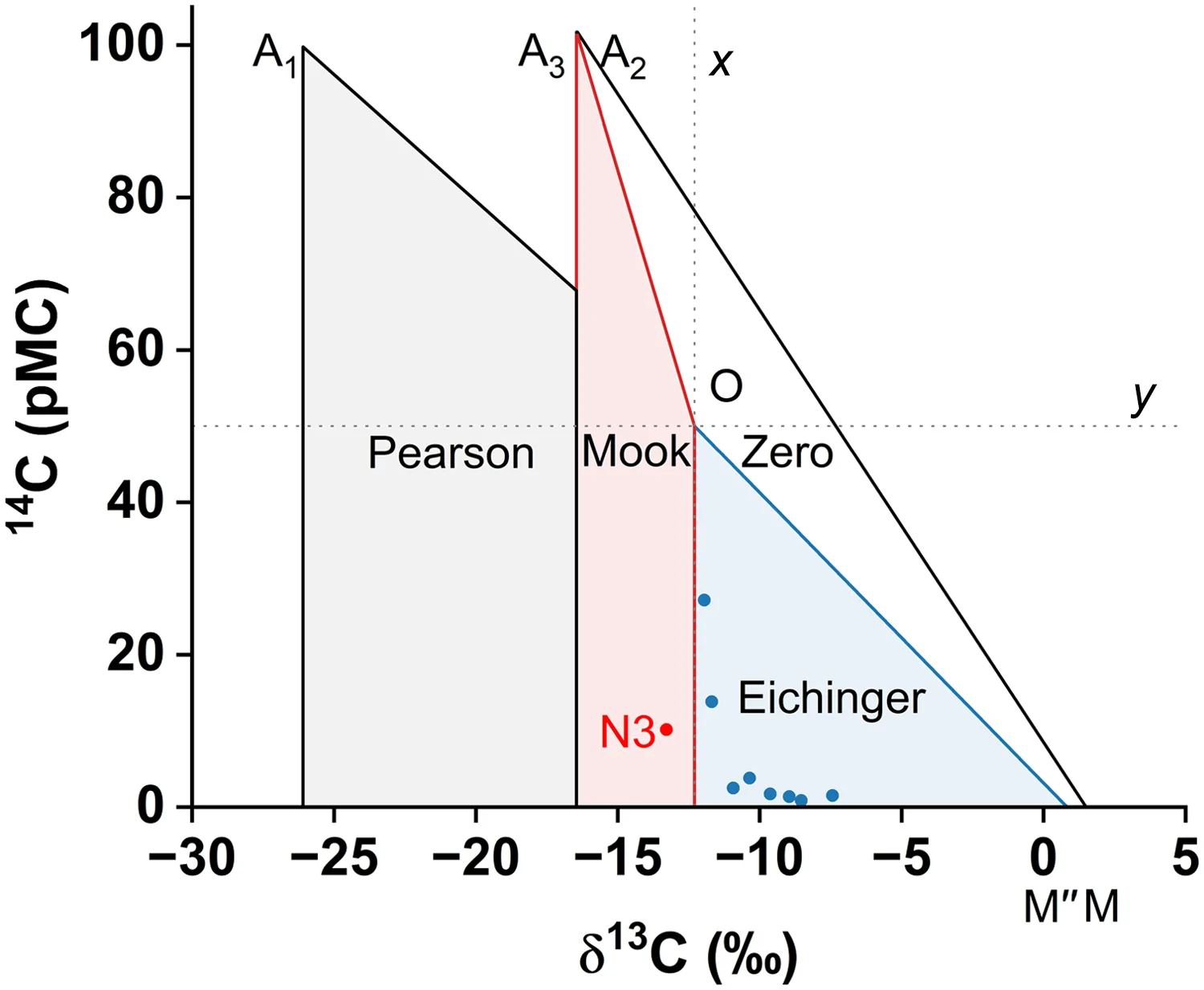
Han-Plummer diagram. Point O denotes the Tamers point. Points A_1_, A_2_, and A_3_ represent the ^14^C and δ^13^C values of dissolved CO_2_, HCO_3_^−^, and DIC after open-system exchange. Point M represents the solid carbonate end-member, and point M″ represents the ^14^C and δ^13^C values of DIC after closed-system carbonate dissolution. The blue-, red-, and gray-shaded areas indicate the applicable regions for the Eichinger, Mook, and Pearson correction models, respectively.

**Table 1.** ^81^Kr and ^14^C results for nine groundwater wells in the North China Plain. The ^81^Kr abundance value of N5 represents the average of two independent measurements.

Sample name	*R*_14_ (pMC)	$R_{14}^{\text{corr}}$ (pMC)	^14^C correction model	*R*_81_ (pMKr)	$R_{81}^{\text{corr}}$ (pMKr)
N1	3.80 ± 0.05	8.99 ± 0.12	Eichinger	93.8 ± 1.0	93.4 ± 1.1
N2	13.81 ± 0.08	29.14 ± 0.17	Eichinger	97.5 ± 1.0	97.3 ± 1.1
N3	10.16 ± 0.07	16.47 ± 0.11	Mook	97.4 ± 1.0	97.2 ± 1.1
N4	2.49 ± 0.05	5.57 ± 0.11	Eichinger	91.1 ± 1.0	90.7 ± 1.0
N5	27.17 ± 0.10	55.95 ± 0.21	Eichinger	89.6 ± 0.7	88.9 ± 0.8
N6	0.86 ± 0.04	2.41 ± 0.11	Eichinger	81.9 ± 0.9	81.5 ± 0.9
N7	1.71 ± 0.05	4.30 ± 0.13	Eichinger	92.0 ± 1.0	91.6 ± 1.0
N8	1.38 ± 0.05	3.71 ± 0.13	Eichinger	54.5 ± 0.8	54.1 ± 0.8
N9	1.51 ± 0.05	4.79 ± 0.16	Eichinger	51.5 ± 0.8	50.1 ± 0.9

To further compare the differences among various ^14^C correction models, we applied commonly used ^14^C correction models to all data, calculated the corresponding ^14^C ages, and compared them with ^81^Kr ages (Table 2). Four of the five models [Tamers, Pearson, Fontes and Garnier, and Eichinger] yielded closely comparable ^14^C ages. The uncertainties reported for the ^14^C ages in the table do not account for uncertainties in the parameters of the correction models. If these parameter uncertainties were included, the ^14^C ages from these four models would be considered consistent. In contrast, the Mook model gave substantially different results. Comparing these ages with ^81^Kr ages reveals that the correction models selected on the basis of the Han and Plummer diagram are appropriate for N1 to N3 and N7. For N4, however, the uncorrected ^14^C age is actually closer to the ^81^Kr age. The reason for this is that all the above models assume closed-system carbonate dissolution in which the carbonate has zero ^14^C content. This assumption lowers the initial ^14^C content, making the corrected ages younger than the uncorrected ones. We infer that the geochemical process corresponding to N4 more closely resembles open-system carbonate dissolution, where the initial ^14^C abundance does not vary greatly (between 100 and 104 pMC), so that the final ^14^C age is very close to the uncorrected value (*29*–*32*). Accordingly, for N1, N2, and N7, we ultimately adopted the ^14^C ages corrected with the Eichinger model; for N3, the Mook model; and for N4, the uncorrected ^14^C age. For the second group of samples, neither the corrected nor uncorrected ^14^C ages are close to the ^81^Kr ages, and we therefore applied no correction to their ^14^C ages.

**Table 2.** ^14^C ages calculated using different correction models. N.A., not applicable.

Sample name	Uncorrected (ka)	Tamers (ka)	Pearson (ka)	Mook (ka)	Fontes and Garnier (ka)	Eichinger (ka)	^81^Kr age (ka)
N1	$27.03_{-0.11}^{+0.11}$	$21.64_{-0.11}^{+0.11}$	$20.38_{-0.11}^{+0.11}$	$14.55_{-0.11}^{+0.11}$	$20.35_{-0.11}^{+0.11}$	$19.91_{-0.11}^{+0.11}$ *	$22.5_{-3.7}^{+3.7}$
N2	$16.37_{-0.05}^{+0.05}$	$11.08_{-0.05}^{+0.05}$	$10.60_{-0.05}^{+0.05}$	$7.94_{-0.05}^{+0.05}$	$10.59_{-0.05}^{+0.05}$	$10.19_{-0.05}^{+0.05}$ *	$9.0_{-3.6}^{+3.7}$
N3	$18.90_{-0.06}^{+0.06}$	$13.27_{-0.06}^{+0.06}$	$14.08_{-0.06}^{+0.06}$	$14.91_{-0.06}^{+0.06}$ *	$14.47_{-0.06}^{+0.06}$	$13.76_{-0.06}^{+0.06}$	$9.4_{-3.6}^{+3.7}$
N4	$30.53_{-0.16}^{+0.17}$ *	$24.86_{-0.16}^{+0.17}$	$24.27_{-0.16}^{+0.17}$	$22.25_{-0.16}^{+0.17}$	$24.25_{-0.16}^{+0.17}$	$23.87_{-0.16}^{+0.17}$	$32.1_{-3.7}^{+3.7}$
N5	$10.77_{-0.03}^{+0.03}$ *	$5.28_{-0.03}^{+0.03}$	$5.16_{-0.03}^{+0.03}$	$3.88_{-0.03}^{+0.03}$	$5.16_{-0.03}^{+0.03}$	$4.80_{-0.03}^{+0.03}$	$38.9_{-3.0}^{+3.0}$
N6	$39.32_{-0.38}^{+0.39}$ *	$33.66_{-0.38}^{+0.39}$	$31.28_{-0.38}^{+0.39}$	$14.27_{-0.38}^{+0.39}$	$31.22_{-0.38}^{+0.39}$	$30.78_{-0.38}^{+0.39}$	$67.6_{-3.5}^{+3.5}$
N7	$33.63_{-0.24}^{+0.25}$	$27.97_{-0.24}^{+0.25}$	$26.46_{-0.24}^{+0.25}$	$20.00_{-0.24}^{+0.25}$	$26.42_{-0.24}^{+0.25}$	$26.02_{-0.24}^{+0.25}$ *	$29.1_{-3.6}^{+3.7}$
N8	$35.41_{-0.29}^{+0.31}$ *	$29.74_{-0.29}^{+0.31}$	$27.72_{-0.29}^{+0.31}$	$18.24_{-0.29}^{+0.31}$	$27.65_{-0.29}^{+0.31}$	$27.23_{-0.29}^{+0.31}$	$203.1_{-5.1}^{+5.2}$
N9	$34.66_{-0.27}^{+0.28}$ *	$28.99_{-0.27}^{+0.28}$	$25.67_{-0.27}^{+0.28}$	N.A.	$25.58_{-0.27}^{+0.28}$	$25.12_{-0.27}^{+0.28}$	$228.4_{-5.8}^{+5.9}$

* Values indicate the final corrected ages selected on the basis of comparison with the ^81^Kr results.

The above discussion demonstrates that combining ^81^Kr with the Han and Plummer approach can substantially reduce the uncertainty in selecting an appropriate ^14^C correction model, help disentangle the complex geochemical processes affecting ^14^C in groundwater, and therefore yield more reliable ^14^C ages. We now proceed to further discuss the ^14^C ages corrected using the approach described above.

## DISCUSSION

### Spatial distribution of groundwater ages and implications for water management

To reveal the spatial evolution of groundwater age along the flow path, we plotted the ^81^Kr and corrected ^14^C ages (hereafter, all ^14^C ages refer to corrected values) as a function of distance from the recharge area in Fig. 4 (also see fig. S6). Over relatively short distances (<90 km), the ^14^C and ^81^Kr ages show consistent trends. Beyond 90 km, where the true groundwater age exceeds the dating range of ^14^C (∼30 ka), the ^14^C ages no longer correlate with distance from the recharge area. In contrast, the ^81^Kr ages continue to increase with distance. This indicates that for deep groundwater in large-scale basin aquifers, the true ages generally fall beyond the ^14^C dating range. From fitting the age-distance relationship, the apparent migration velocities derived from ^14^C and ^81^Kr are ∼2 and 0.2 m/year, respectively (see fig. S7). The apparent migration velocities differ by nearly an order of magnitude, with ^81^Kr indicating substantially slower renewal of the deep confined aquifer. Thus, incorporating ^81^Kr dating into groundwater studies can effectively determine whether ^14^C remains applicable, enable reasonable estimation of flow direction and velocity, and avoid overestimation of groundwater renewal rates.

**Fig. 4.**
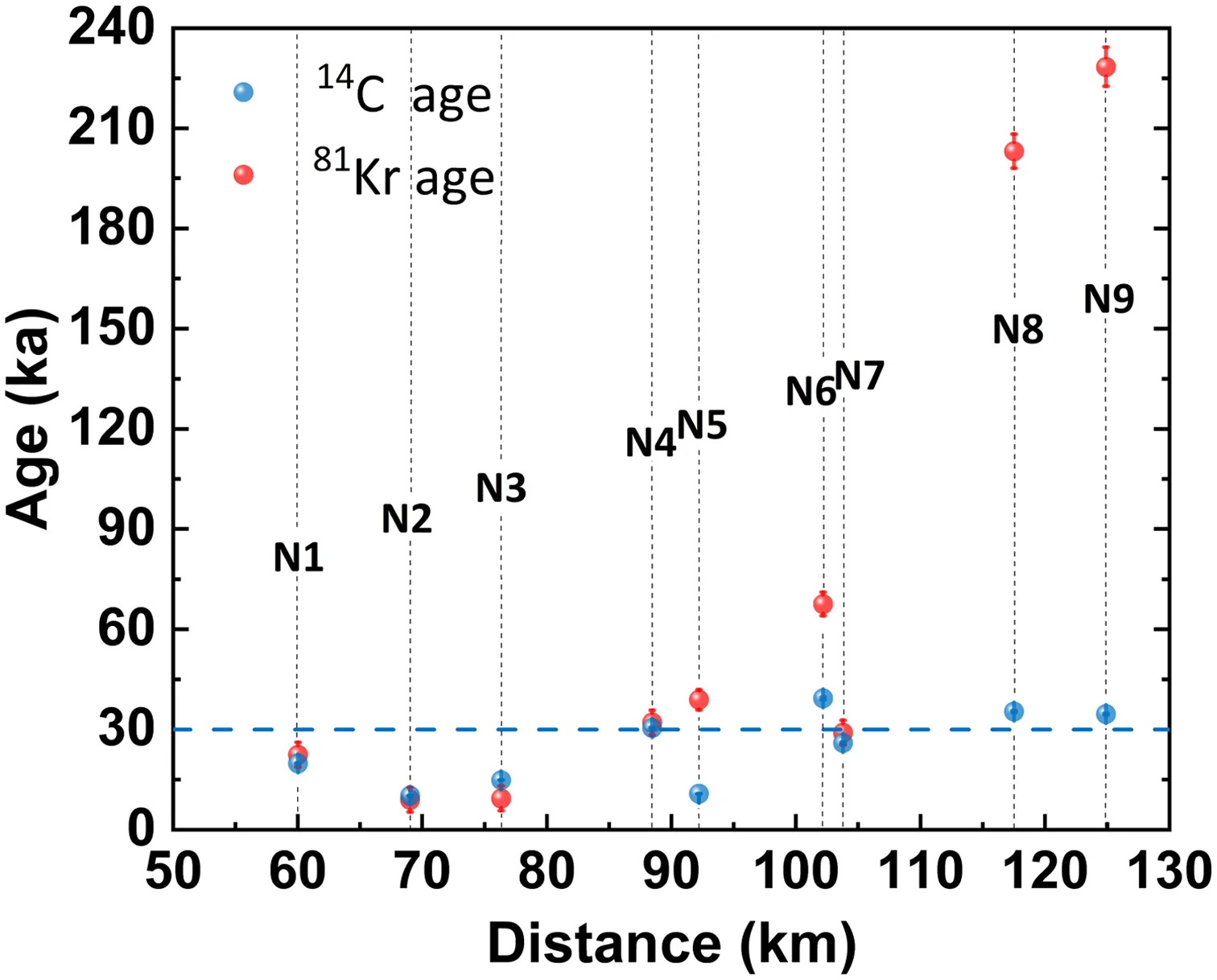
Groundwater age as a function of distance from the recharge area. ^81^Kr ages (red dots) exhibit a trend with the increasing distance, while ^14^C ages (blue dots) reach their limit at ∼30 ka (blue dashed line).

Current groundwater management in the North China Plain relies primarily on an integrated strategy including delineation of overexploitation zones, control of total abstraction and groundwater level, alternative water supply allocation, abstraction reduction, and artificial recharge (*33*). Groundwater ages can provide key constraints on the renewability and associated timescales of deep aquifers. The results have clear implications for resource management: Short-term groundwater level recovery does not equate to rapid natural renewal of deep, ancient groundwater resources. Instead, deep ancient groundwater should be considered a strategic resource that renews very slowly. Management priorities should focus more on long-term extraction limits and intergenerational resource protection, rather than treating it as conventional groundwater that can be naturally replenished over short cycles.

### Influence of groundwater mixing and dispersion

In previous sections, we discussed how ^81^Kr data can be used to select appropriate ^14^C correction models and examined the relationship between apparent ages and recharge distance for both tracers. For most samples within the ^14^C dating limit (30 ka), specifically N1 to N4, and N7, the ^14^C ages are consistent with the ^81^Kr ages, suggesting that groundwater flow behaves as the classical piston flow model. In contrast, although the ^14^C age of N5 remains within its measurement limit, it deviates markedly from the apparent ^81^Kr age. The young apparent ^14^C age coupled with the older apparent ^81^Kr age indicates the influence of groundwater mixing or dispersion.

We first consider the simplest case: a two-end-member mixing model. Assuming that one end-member is modern groundwater ($R_{14}=100\;\text{pMC}$ and $R_{81}=100\;\text{pMKr}$), we calculate that the old end-member has a groundwater age of 55 ka ($R_{14}=0.14\;\text{pMC}$ and $R_{81}=85\;\text{pMKr}$), with a mixing ratio of 27%:73% between the young and old end-members (see the “Two-end-member mixing calculation” section in the Supplementary Materials).

In addition to mixing between young and old groundwater, dispersion during transport can also produce a distribution of groundwater ages, causing deviation from the single age expected from the piston flow model and leading to contrasting apparent ages for tracers with different time responses. To explore this possibility, we used TracerLPM to simulate piston flow and dispersion models with various parameters (see fig. S8). The results indicate that N1 to N4 and N7 are closer to the piston flow model, whereas N5 is consistent with neither the piston flow model nor dispersion models with different parameters. Therefore, the most plausible explanation for the deviation of N5 from the piston flow model is mixing between different end-members, rather than dispersion during groundwater transport.

The example of sample N5 highlights the importance of combining ^81^Kr and ^14^C in groundwater studies. Previous studies have often been unable to identify groundwater samples affected by dispersion or mixing processes. In such samples, paleoclimate proxies (e.g., δ^18^O and noble gas temperatures) reflect averaged signals rather than the climatic conditions at the time of recharge, potentially introducing systematic biases into paleoclimate reconstructions. The combined use of ^81^Kr and ^14^C can address this issue to a large extent by leveraging their cross-comparison to identify groundwater that has been influenced by dispersion, mixing, or modern contamination, thereby excluding unsuitable data from paleoclimate reconstructions.

### Paleoclimate information from groundwater based on the ^81^Kr and ^14^C age framework

Synthesizing the discussions above, the integration of ^81^Kr and ^14^C dating not only establishes a reliable groundwater age framework but also enables the screening of groundwater samples suitable for paleoclimate reconstruction. This substantially improves the quality of groundwater data and is critical for correctly extracting and interpreting paleoclimate information.

Following this approach, we showed that among the nine groundwater samples, only N5 was affected by mixing, whereas the remaining samples can be further used for paleoclimate proxy interpretation. Therefore, the stable water isotope data of all samples were analyzed except N5. The results show that their δ^18^O and δD values fall on the local meteoric water line parallel to the global meteoric water line (see fig. S9). This indicates that these groundwater samples have not been substantially affected by water-rock interactions, thus preserving the paleoclimate information from the time of recharge. We further compared the δ^18^O data obtained in this study with those from a neighboring study area reported by Kreuzer *et al.* (*34*), who used ^14^C dating to cover groundwater ages over the past 30 ka. The results show that within the same age range, the δ^18^O trends and ranges of our samples are similar to those of the previous study (Fig. 5).

**Fig. 5.**
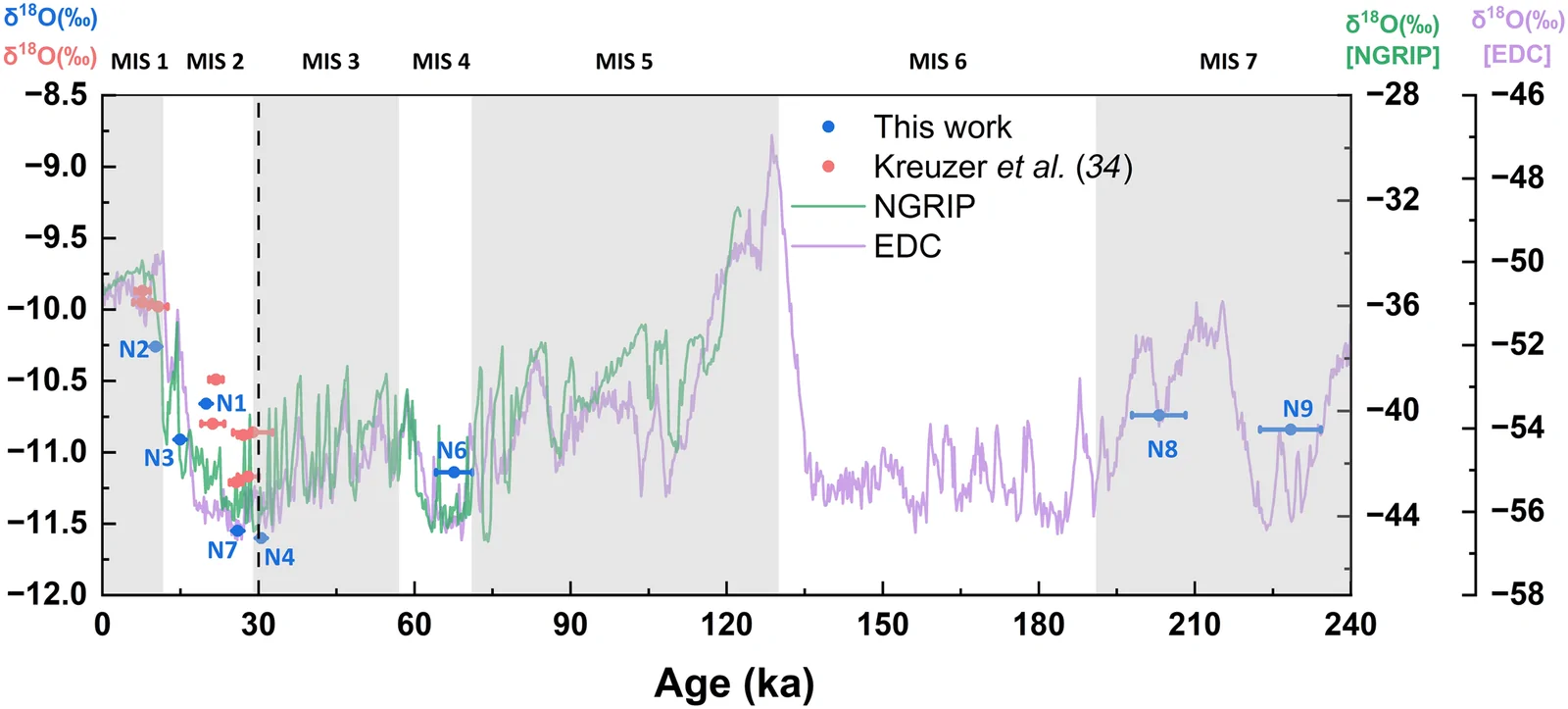
Groundwater δ^18^O records from North China Plain compared with polar ice core records. Blue dots represent data of this work, and the red dots are data from Kreuzer *et al.* (*34*). The green line shows data of the NGRIP ice core in Greenland (*35*), and the purple line represents data from the EDC ice core in Antarctica (*36*). Both ice core records are smoothed over 100-year intervals (*37*). With the precise ^81^Kr ages, groundwater recharge events can be attributed to glacial or interglacial periods (shaded areas). Samples N1, N2, N3, N4, and N7 are plotted using corrected ^14^C ages, whereas samples N6, N8, and N9 are plotted using ^81^Kr ages.

We further compared the groundwater δ^18^O data with δ^18^O records from the NGRIP (North Greenland Ice Core Project) and EDC (EPICA Dome C) ice cores (*35*–*37*). For samples with ages of ∼30 ka or younger, we used the corrected ^14^C ages selected on the basis of high-precision ^81^Kr dating. For samples substantially older than 30 ka, we used the ^81^Kr ages. We found that in the time window within 30 ka, the data from this study show better consistency with the ice core records than do the data from Kreuzer *et al.* (*34*). This indicates that the data filtered by the combined ^81^Kr and ^14^C approach can more accurately reflect paleoclimatic conditions. Furthermore, ^81^Kr enables the reconstruction of paleoclimate records on timescales beyond the dating range of ^14^C. Samples N8 and N9 both formed during Marine Isotope Stage 7 (MIS 7), and their δ^18^O responses are consistent with the EDC ice core record from the Southern Hemisphere. This demonstrates that the δ^18^O signal in precipitation over the North China Plain reflects global-scale temperature variations rather than regional climatic information.

### Implications for Last Glacial Maximum studies

The Last Glacial Maximum (LGM), as the most recent global extreme cold event, holds an important position in paleoclimate research (*38*–*43*). During this period, continental ice sheets expanded, sea levels fell, and greenhouse gas concentrations decreased, providing an important reference for testing the response of the climate system to external forcing. Therefore, accurately characterizing the LGM—particularly climate responses in the mid-to-low latitudes—is critical for deepening our understanding of past climate mechanisms and improving the reliability of future climate projections.

Previous studies have made important advances in using ancient groundwater to investigate the LGM. For example, Seltzer *et al.* (*42*) compiled noble gas temperature data from multiple groundwater studies across different regions to estimate the cooling magnitude at the LGM relative to the Late Holocene (*42*). Specifically, for samples within the 15- to 45-ka range, the study first identified data points with the lowest temperatures, then included all candidate samples within 1°C of this minimum value, and lastly calculated a weighted average of this sample set to represent the temperature during the LGM. However, the groundwater ages in these studies were mostly determined using the ^14^C method, which may introduce bias into sample selection because of the issues discussed above. First, ^14^C ages obtained from different correction models vary considerably, and the choice of model involves substantial uncertainty, which limits the consistency of groundwater ages across different studies. Second, relying on a single tracer makes it difficult to identify complex processes such as groundwater mixing or dispersion during transport. Consequently, a set of ancient groundwater samples selected solely on the basis of ^14^C ages may include samples affected by these processes, compromising the accuracy of LGM temperature reconstructions. These problems can be effectively mitigated by the ^81^Kr-^14^C dual-tracer approach introduced in this work.

Furthermore, the study by Seltzer *et al.* (*42*) only included data from latitudes between 45°S and 35°N. This approach was primarily intended to exclude the influence of ice-sheet proximity effects on groundwater north of 35°N. However, during the LGM, large Northern Hemisphere ice sheets, such as the Laurentide and Fennoscandian ice sheets, were centered over North America and northern Europe, whereas much of interior northern Asia, including northern China, remained free of continental ice-sheet cover (*44*–*46*). Therefore, ancient groundwater in parts of northern Asia north of 35°N may still preserve relatively intact paleoclimate information. The groundwater data from the North China Plain reported in this work support this notion, as their δ^18^O records show good consistency with ice core records. The inclusion of additional groundwater data from northern Asia could further improve the representativeness of the data and the accuracy of global mean temperature estimates for the LGM.

## MATERIALS AND METHODS

### Age uncertainty calculation in ^81^Kr dating

For ^81^Kr dating, the corresponding age uncertainty can be calculated as follows

$$\delta t=\frac{t_{1/2}}{\text{ln}\;2}\cdot \frac{\delta R_{81}}{R_{81}}$$ (2)

where δt is the age uncertainty, $t_{1/2}$ is the half-life of ^81^Kr, $R_{81}$ is the ^81^Kr abundance relative to the modern level, and ${\delta R}_{81}$ is the uncertainty in $R_{81}$.

Previous studies using ATTA systems achieved typical analysis errors at 5% of the modern ^81^Kr abundance. According to Eq. 2, for samples younger than 50 ka, the 5% analysis error translates to an age uncertainty of ±18 ka, leading to a lower age limit of 50 ka (approximately three times the standard deviation) for ^81^Kr dating. In other words, ^81^Kr dating has previously been unable to determine ages younger than 50 ka.

### High-precision ATTA system

To achieve high-precision measurements of ^81^Kr abundance, the ATTA instrument must meet several requirements. First, the count rate of ^81^Kr must be sufficiently high to ensure adequate statistical precision within the limited measurement time. Second, the measurement precision for the reference stable isotope must also be better than 1%. Last, because high-precision measurements require long acquisition times, the optical system of the instrument must remain stable over extended periods of operation—including optical power, optical polarization, and beam pointing. To satisfy these requirements, we integrated a magnetically enhanced radio-frequency discharge source (*47*), a high-precision ion-current measurement method for the atom loading rate (*48*), and a fiber-based modular optical system into the high-precision ATTA system (see the “Instrumentation improvements for high-precision ATTA” section in the Supplementary Materials for details).

An external magnetic field–enhanced radio-frequency discharge source capable of producing an intense metastable Kr atomic beam was used in the ATTA system. The design follows Yan *et al.* (*47*). By adding permanent magnets to provide magnetic field confinement of the plasma, a higher metastable production efficiency is achieved. For a modern atmospheric sample, the ^81^Kr count rate reaches 6000 atoms/hour, reducing the statistical uncertainty of atom counting to below 1% over a 3-hour measurement period. For the stable reference isotope, we used an ion current monitor method to measure the trap loading rate of ^83^Kr (isotopic abundance: 11.5%) (*48*). Under steady-state conditions, the cold collision between trapped ^83^Kr atoms is the dominant loss channel, resulting in Penning ionization and associative ionization of the atoms. The ions are directed to and collected by a Faraday cup. The recorded ion current is a precise indicator of the trap loading rate. Last, an all-fiber–coupled optical design was used. As a result, the drifts resulting from temperature changes have been reduced substantially, improving the stability of the atom trap and the signal-to-noise ratio in single-atom detection (see fig. S2C).

By combining the technical measures described above, we ultimately achieved a measurement uncertainty of less than 1% for the ^81^Kr abundance in modern samples, corresponding to an age uncertainty of ±3 ka (see Eq. 2). The lower limit of ^81^Kr dating has been extended to 10 ka.

### Calibration of the ATTA instrument

To validate that the method meets the required precision, we prepare a series of standard samples for calibration measurements. However, producing standards of ^81^Kr is practically challenging because of the lack of a blank for this isotope with such a long half-life. As an alternative, we verify the measurement precision using the other rare isotope of krypton, ^85^Kr (half-life = 10.74 a), relying on the high similarity between the two isotopes. ^85^Kr in the atmosphere is anthropogenic and has an isotopic abundance of ^85^Kr/Kr = 1 × 10^−11^ to 2 × 10^−11^, only one order of magnitude higher than that of ^81^Kr (*19*). We prepare nine standard samples with varying ^85^Kr/Kr ratios by precisely mixing a modern ^85^Kr sample with a blank. The modern ^85^Kr sample is from a commercial krypton gas purchased in 2018. Its ^85^Kr/Kr (activity) was determined to be 57.7 ± 1.2 dpm/cm^3^ in February 2023 and defined in this work as 100% [here, the traditional unit dpm/cm^3^ stands for decays per minute per ml STP (standard temperature and pressure) of krypton]. The blank was collected at the University of Bern before the dawn of anthropogenic nuclear activities. Its ^85^Kr/Kr (activity) was determined to be less than 1 dpm/cm^3^ by low-level decay counting in 2011 (*21*). Using the high-precision ATTA system, we directly measured its abundance to be (0.72 ± 0.05)% of the modern ^85^Kr sample. For each standard sample, its ^85^Kr/Kr ratio is determined on the basis of the gas mixing ratios, reaching a precision at 0.2% of the modern level (see the “Mixing system” section and fig. S3 in the Supplementary Materials).

We measure the nine standard samples using the high-precision ATTA system, with each measurement consuming ∼10 μl STP of krypton. The ATTA-measured results are compared to the expected ^85^Kr/Kr ratios (see fig. S4). A linear fit of the data points reveals a proportional relationship between the measured and expected ^85^Kr/^83^Kr ratios ($\chi^{2}=0.6$). The comparison confirms that the system has achieved a precision level at 1% of the modern abundance level.

### Input correction for the ^81^Kr abundance

The initial ^81^Kr abundance, $R_{81}^{\text{init}}$, can be determined on the basis of the ^81^Kr input curve in the atmosphere (fig. S5) (*19*), which is generated by integrating a cosmic-ray flux model, historical geomagnetic field data, and isotope decay laws over time (*19*).

Last, the corrected content of ^81^Kr is

$$R_{81}^{\text{corr}}=\frac{R_{81}}{R_{81}^{\text{init}}}$$ (3)

The details are provided in the Supplementary Materials (see the “Correction for ^81^Kr” section in the Supplementary Materials).

### Correction for the ^14^C content

The correction process for ^14^C content follows the procedure outlined by Han and Plummer (*28*). First, on the basis of pH, temperature, alkalinity values, and major inorganic chemistry, the concentrations of total DIC, ${\text{HCO}}_{3}^{-}$, ${\text{CO}}_{3}^{2-}$, and ${\text{CO}}_{2}$ in the sample are calculated using PHREEQC software (see tables S2, S3, and S5). These data are then used to plot the Han-Plummer diagram. Using the USGS Netpath program, the initial ^14^C content could be determined, denoted as $R_{14}^{\text{init}}$ (table S7, Supplementary Materials). The selection of different model outputs from Netpath is based on the Han-Plummer diagram. The δ^13^C value of soil-derived ${\text{CO}}_{2}$ is assumed to be −25‰ (a commonly used value for temperate regions) (*22*), while the δ^13^C value of solid carbonate minerals is taken as 1.5‰ (*34*). Last, the corrected ^14^C content $R_{14}^{\text{corr}}$ is given by

$$R_{14}^{\text{corr}}=\frac{R_{14}}{R_{14}^{\text{init}}}$$ (4)

The details are provided in the Supplementary Materials (see the “Correction for ^14^C” section in the Supplementary Materials).

### Groundwater sampling

Site access for this study was coordinated by the Institute of Hydrogeology and Environmental Geology. Samples were collected from preexisting wells with the assistance of the well managers, and no new wells were drilled for this study. As these wells had been drilled under previously obtained permits and are routinely sampled by the Institute of Hydrogeology and Environmental Geology for conventional water-quality monitoring, no specific permits were required.
