# Supplementary material for: High-precision 81Kr dating overcomes radiocarbon limits in Late Pleistocene groundwater
Source: Sci Adv. 2026 Nov 13;12(46):eaef6864. doi: 10.1126/sciadv.aef6864 (PMC13763926; doi:10.1126/sciadv.aef6864)
Supplement: Supplementary file 1 [file sciadv.aef6864_sm.pdf]

Science Advances
AAAS

Supplementary Materials for

High-precision ^{81}Kr dating overcomes radiocarbon limits in Late Pleistocene groundwater

Hao Li *et al.*

Corresponding author: Wei Jiang, wjiang1@ustc.edu.cn; Zheng-Tian Lu, ztlu@ustc.edu.cn

Sci. Adv. **12**, eaef6864 (2026)
DOI: 10.1126/sciadv.aef6864

This PDF file includes:

Supplementary Text
Figs. S1 to S9
Tables S1 to S8

Atom Trap Trace Analysis (ATTA)

Based on laser manipulation of neutral atoms, ATTA selectively capture and detect individual atoms of the desired isotope *(21)*. It achieves the required sensitivity, selectivity, and efficiency by employing multiple techniques in atom optics, laser trapping and cooling, among which the magneto-optical trap (MOT) plays a central role. The detection process involves multiple stages: metastable krypton atoms are first generated through electron collisions, followed by transverse cooling and focusing to reduce radial velocity spread. The atoms are then slowed down in the longitudinal direction via a Zeeman decelerator. Finally, the atoms are captured in a three-dimensional MOT, where they undergo repeated photon scattering. A highly sensitive electron-multiplying charge-coupled device (EMCCD) camera collects the fluorescence signals, enabling single-atom detection.

Age is calculated based on the measured isotopic abundances:

$$R_{81} = \frac{\left[{}^{81}\mathrm{Kr}/{}^{83}\mathrm{Kr}\right]_{sample}}{\left[{}^{81}\mathrm{Kr}/{}^{83}\mathrm{Kr}\right]_{atmosphere}} = 2^{-\frac{Age}{Half-life}} \qquad (S1)$$

Instrumentation improvements for high-precision ATTA

1. External-magnetic-field-enhanced Radio-Frequency discharge source (Fig. S2A)

To reduce statistical errors in atom counting, we used an external-magnetic-field-enhanced radio-frequency (RF) discharge source to produce an intense beam of metastable Kr (Kr*) atoms. The design of the source largely follows the one reported by Yan *et al.* 2023 *(47)*. Modest changes were made to optimize its performance, including a longer permanent magnet that provides stronger B-field and a quartz shield covering the magnetic shield to reduce metal sputtering.

The design has an external B-field to provide more confinement to the plasma in the Radio-Frequency discharge Kr* source. This increases the interaction time between the electrons and the background Kr gases, thus a higher Kr* production efficiency. Several things need to be considered in order to meet the requirements for the ATTA application. (1) The source needs to be high vacuum compatible so that it can be placed inside the vacuum chamber. (2) In order to produce a cold Kr* beam, the central tube confining the working gas needs to have a high thermal conductivity so that it can be cooled to low temperature (<130 K). (3) A magnetic shield is needed so that the stray B-field does not interfere with the atomic optics (transverse cooling) close to the exit of the discharge source. A permanent ring magnet is used to generate the external B-field needed for plasma confinement. To suppress the stray B-field leaking out of the source, μ-metal (1J50, saturation magnetic induction intensity 1.5 T) shields with a thickness of 10 mm are installed around the magnet.

The B-field inside the discharge source can reach 900 Gs, which is adequate for the plasma confinement. Outside the source, the B-field decreases rapidly to less than 20 Gs within 20 mm from the exit of the AlN (Aluminum nitride) tube. As the transverse cooling can tolerate a stray B-field as high as 25 Gs, the leakage B-field from the discharge source does not affect the normal operation of the transverse cooling stage in ATTA.

The new Kr* source has a beam intensity 2 to 4 times higher than the old Kr* source, depending on the working conditions.

2. Ion current method (Fig. S2B)

An ion current monitor method was used to measure the trap loading rate of ^{83}Kr *(48)*. The ion current method is employed due to its high precision and stability. The basic principle of this method is as follows. The number of ^{83}Kr atoms in the atom trap is governed by the following equation:

$$\frac{dN(t)}{dt} = L - \frac{N(t)}{\tau} - \beta N^2(t) \quad (S2)$$

$N(t)$ is the number of atoms, L is the loading rate, τ is the lifetime of metastable atoms, β is the collision parameter between metastable atoms.

The last two terms on the right-hand side of the equation represent the loss terms for the atoms in the trap. The first term accounts for the loss of metastable krypton atoms due to collisions with background gases. The second term represents the loss due to two-body collisions between metastable krypton atoms in the trap. The two-body collisions include the Penning ionization and associative ionization process.

Penning ionization:

$$\mathrm{Kr}^* + \mathrm{Kr}^* \rightarrow \mathrm{Kr} + \mathrm{Kr}^+ + \mathrm{e}^- \quad (S3)$$

Associative ionization:

$$\mathrm{Kr}^* + \mathrm{Kr}^* \rightarrow \mathrm{Kr}_2^+ + \mathrm{e}^- \quad (S4)$$

Since both processes produce singly charged ions, they are the same in terms of ion current production and cannot separate from each other. Under normal operating conditions, the two-body collision loss is the dominant loss channel for the Kr* atoms in the trap. So, in steady state, the loading rate of the atom trap (L) is proportional to the ion current produced from the trap, $L = \beta N^2(t)$. This signal is detected in the ATTA measurement as a precise measure of the loading rate of the reference isotope.

Fig. S2B is the schematic of the ion current collection device and the simulation of ions trace. The design follows the one reported by Jiang *et al.* 2014 *(48)*. Four semi-circular electrodes are symmetrically positioned in pairs around the atom trap with a Faraday cup placed on one side for ion collection. By applying appropriate voltages to the four electrodes, ions produced in the trap are deflected into the Faraday cup, enabling the detection of the ion current signal. Based on this scheme the loading rate of the reference isotope can be measured with a precision better than 0.5%.

3. All fiber-coupled optics (Fig. S2C)

To make the systematic effects due to the slow drifts of the optics under control, an all-fiber coupled optics design has been implemented. This design removed many free-space optical elements in the old optical layout and increased the long-term stability. The fiber coupling scheme also allows decoupling of optical modules with different functions. As a result, the drifts due to temperature changes have been reduced substantially, which is particularly important for high precision measurements with long measurement time. Finally, the qualities of the light beam used for the atom trap are improved by using single-mode fibers, which lead to more stable confinement of the atom and a better signal-to-noise ratio in single atom detection.

Mixing system

We mix two samples with different proportions to produce a total of nine standard samples of ^{85}Kr. One is a modern sample whose ^{85}Kr relative abundance is designated to be $C_1 = 100\%$, the other is a blank sample ($C_2 = 0.72\%$). Mixing is carried out in an apparatus shown in Fig. S3 (Supplementary) with the following procedure. First, the Buffer Tank is filled to a pressure of P_1 with the modern sample. The pressure is measured with a capacitance manometer to an accuracy of 0.12%. The gas is then transferred from the Buffer Tank to the sample holder, reducing the Buffer Tank pressure down to P_1'. Therefore, the amount of gas transferred from the Modern holder

to the Standard holder is $(P_1 - P_1') \cdot V_{Buffer}$. Subsequently, an amount of gas equal to $(P_2 - P_2') \cdot V_{Buffer}$ is transferred from the blank holder to the Standard holder.

The relative abundance of the standard sample C_m can be calculated as follows:

$$C_m = \frac{C_1 \cdot (P_1 - P_1') \cdot V_{Buffer} + C_2 \cdot (P_2 - P_2') \cdot V_{Buffer}}{(P_1 - P_1') \cdot V_{Buffer} + (P_2 - P_2') \cdot V_{Buffer}} = \frac{C_1 \cdot (P_1 - P_1') + C_2 \cdot (P_2 - P_2')}{(P_1 - P_1') + (P_2 - P_2')} \quad (S5)$$

The relative abundances of the standard samples are shown in Table S1.

Correction for ^{81}Kr

For cases of $R_{81} < 1$, the sample is too old for any original ^{85}Kr to be present, thus the ^{85}Kr decay activity (denoted R_{85}) is expected to be zero. Any presence of ^{85}Kr indicates a mixture of young or modern gas sources, and a correction must be made in the age calculation. The contamination can come from different sources: 1) Mixing with young groundwater (age < 50 a). Additional information about the source of mixture is needed to obtain the ages of different end members. 2) Mixing with air during the gas extraction and purification process. Air can be introduced into the groundwater and contaminate the gas sample in the well head or in the pumping system. In addition, leaks can occur in the degassing system or the sample bottle.

For case 2), effects of air contamination can be corrected. The relationship between the ^{85}Kr-corrected ^{81}Kr abundance (R_{81}') and the measured ^{81}Kr abundance (R_{81}), both relative to the modern air value, is

$$R_{81} = R_{81}' \cdot (1 - \eta) + \eta \quad (S6)$$

where η is the fraction of the krypton coming from modern contaminants. The contamination fraction η can be extracted using the measured ^{85}Kr activity (R_{85}) and the ^{85}Kr activity of the local air at the sampling site (R_{85_air}):

$$R_{85} = R_{85\_air} \cdot \eta \quad (S7)$$

By combining Eq. S6 and S7, the ^{85}Kr-corrected ^{81}Kr abundance (R_{81}') can be calculated. The corrected values are reported in the R_{81}' column of Table S4.

^{81}Kr is a cosmogenic isotope. Its production rate is directly related to the flux of the incoming cosmic ray particles. Since the cosmic ray intensity on earth varies with time, the ^{81}Kr abundance in the atmosphere is not a constant. Based on the relative geomagnetic field intensities recorded over the past 2 Ma the atmospheric ^{81}Kr/Kr abundance has been calculated in Zappala et al *(19)*. During the Late Pleistocene, variations in the initial atmospheric ^{81}Kr abundance are less than 0.5%. The ^{85}Kr-corrected ^{81}Kr abundances are projected onto the atmospheric input-function curve to determine the corresponding initial ^{81}Kr abundance for each sample, denoted as R_{81}^{init} (Fig. S5). Finally, the corrected abundance of ^{81}Kr is:

$$R_{81}^{corr} = \frac{R_{81}'}{R_{81}^{init}} \quad (S8)$$

The results after each correction step are summarized in Table S4.

Correction for ^{14}C

The radiocarbon correction of dissolved inorganic carbon (DIC) was performed following the graphical and process-based approach proposed by L.F. Han and L.N. Plummer *(28)*. In this approach, the measured ^{14}C, $\delta\,^{13}C$, and DIC speciation data are evaluated together to identify the dominant carbon-evolution process and to select an appropriate single-sample correction model. Han and Plummer recommend using the graphical relationship between ^{14}C and $\delta\,^{13}C$ to distinguish among process-based correction models.

First, the aqueous carbonate speciation of each groundwater sample was calculated from the measured pH, temperature, alkalinity, and major-ion chemistry using PHREEQC (Table S2 and S3). The calculated concentrations of TDIC, HCO_3^-, CO_3^{2-}, and CO_2(aq) for all samples are reported in Table S5. For the Han-Plummer graphical calculation, the carbon-species weighting was based on:

$$C_T = C_a + C_b \tag{S9}$$

where C_a is the concentration of CO_2 (aq), and C_b is the concentration of HCO_3^-. This definition follows the simplified DIC framework used in the Han-Plummer formulation, in which CO_2 (aq) and HCO_3^- are treated as the dominant dissolved inorganic carbon species for the isotope mass-balance calculation. The full TDIC value calculated by PHREEQC may include additional inorganic carbon species or ion pairs, but the Han-Plummer diagram calculation uses $C_T = C_a + C_b$ for the concentration of TDIC.

Second, the relevant isotope end-member values were defined for the studied aquifer system. The ^{14}C activity of soil CO_2 was assumed to be 100 pMC, and its $\delta\ ^{13}C$value was set to -25‰, a commonly used value for temperate-zone soil CO_2 dominated by C3 vegetation. The carbonate-mineral end member was assigned a ^{14}C activity of 0 pMC and a $\delta\ ^{13}C$ value of +1.5‰. These end-member values, together with the measured sample temperature and carbonate speciation, were used to calculate the characteristic points A1, A2, A3, O, M, and M''. The calculated coordinates were then used to construct a Han-Plummer ^{14}C- $\delta\ ^{13}C$ diagram for the groundwater system.

The initial value of radiocarbon was performed using the single-sample correction models implemented in NETPATH. The soil CO_2 end member was assigned ^{14}C=100 pMC and $\delta\ ^{13}C$=-25‰, whereas the carbonate mineral end member was assigned ^{14}C=0 pMC and $\delta\ ^{13}C$=+1.5‰. NETPATH was then used to calculate the corrected initial radiocarbon activity R_{14}^{init}, for each sample using its built-in single-sample models, including Vogel, Tamers, Ingerson and Pearson, Mook, Fontes and Garnier, Eichinger models. The most appropriate correction model was selected by comparing the measured ^{14}C–$\delta\ ^{13}C$ values with the Han–Plummer diagram, and the selected initial value R_{14}^{init} was used to calculate the corrected age.

Finally, the corrected ^{14}C content R_{14}^{corr} is given by:

$$R_{14}^{corr} = \frac{R_{14}}{R_{14}^{init}} \tag{S10}$$

Two-end-member mixing calculation

Assuming that sample N5 represents a two-end-member mixture, and that the young end member is modern groundwater, the age of the old end member and the mixing fraction can be estimated from the equations for ^{14}C and ^{81}Kr. The isotope abundances of the mixed sample are expressed as:

$$a \cdot R_{14\_1} + (1 - a) \cdot R_{14\_2} = R_{14}^{corr} \tag{S11}$$

$$a \cdot R_{81\_1} + (1 - a) \cdot R_{81\_2} = R_{81}^{corr} \tag{S12}$$

where a is the fraction of the young groundwater end member, and $(1 - a)$ is the fraction of the old groundwater end member. R_{14_2} and R_{81_2} are the ^{14}C and ^{81}Kr abundances of the old end member, respectively. The modern end member is assumed to have $R_{14\_1} = 100\ pMC, R_{81\_1} = 100\ pMKr$. The ^{14}C and ^{81}Kr abundances of the old end member are constrained to correspond to the same groundwater age:

$$-\frac{t_{1/2\_14}}{\ln 2} \cdot \ln R_{14\_2} = -\frac{t_{1/2\_81}}{\ln 2} \cdot \ln R_{81\_2} \tag{S13}$$

where $t_{1/2\_14}$ and $t_{1/2\_81}$ are the half-lives of ^{14}C and ^{81}Kr. For sample N5, the corrected isotope abundances are $R_{14}^{corr} = 27.17\ pMC, R_{81}^{corr} = 88.9\ pMKr$. Solving Eqs. S11-S13 gives an old end

member with an apparent groundwater age of approximately 55 ka, corresponding to $R_{14\_2} = 0.14\ pMC, R_{81\_2} = 85\ pMKr$. The resulting mixing fraction is approximately 27% modern groundwater and 73% old groundwater.

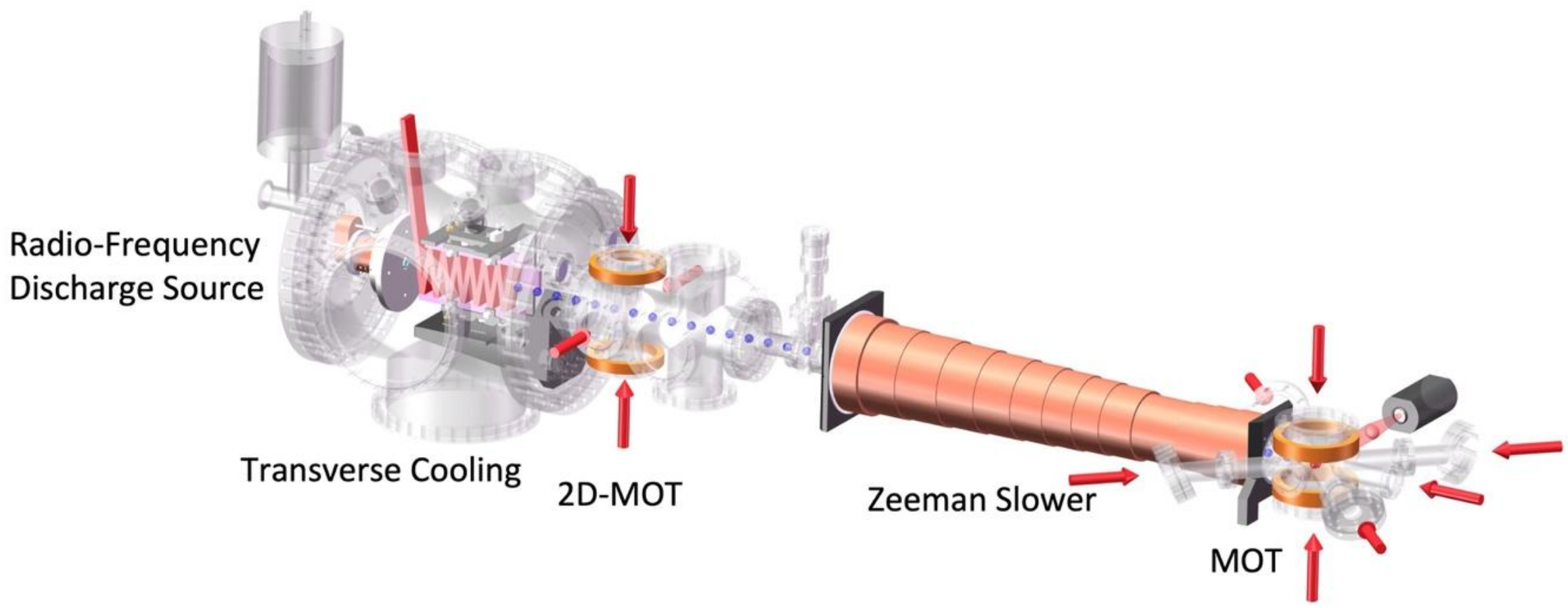

Fig. S1. Atom Trap Trace Analysis apparatus. Metastable krypton atoms generated in the radio-frequency discharge source are first collimated by transverse cooling and then captured and further cooled in the two-dimensional magneto-optical trap (2D-MOT). The atomic beam is subsequently decelerated by the Zeeman slower and delivered to the final three-dimensional magneto-optical trap (MOT), where krypton atoms are selectively trapped and detected by laser-induced fluorescence. Red arrows indicate the main laser-beam directions used for cooling, slowing, and trapping. The figure was created by Zhuoling Precision Equipment (Hefei) Co., Ltd using SolidWorks and is reproduced with permission.

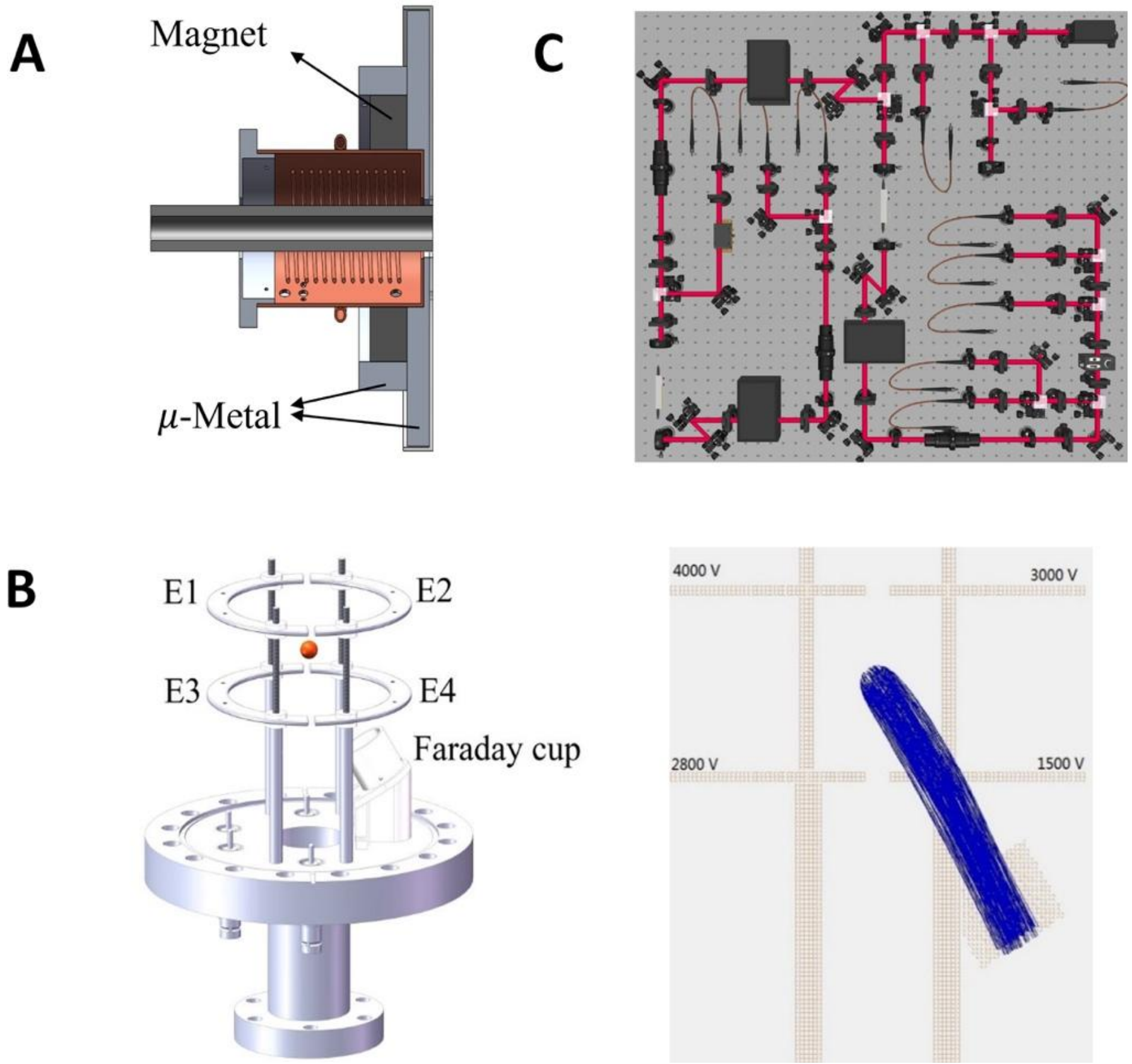

Fig. S2. Instrumentation improvements for high-precision ATTA measurements. (**A**) Magnetically enhanced radio-frequency (RF) discharge source for increasing the production efficiency of metastable krypton atoms. A permanent ring magnet provides magnetic-field confinement of the plasma, while μ-metal shielding suppresses stray magnetic fields near the atomic-beam exit. The figure was created by Zhuoling Precision Equipment (Hefei) Co., Ltd using SolidWorks and is reproduced with permission. (**B**) Ion-current detection scheme for monitoring the loading rate of the reference isotope. The left panel shows the ion-collection apparatus, in which ions produced by Penning and associative ionization in the atom trap are guided by electrodes and collected by a Faraday cup. The figure was created by Zhuoling Precision Equipment (Hefei) Co., Ltd using SolidWorks and is reproduced with permission. The right panel shows the simulated electric-field distribution used to guide ions from the trap region to the Faraday cup. (**C**) All-fiber-coupled optical layout used to improve long-term optical stability and reduce systematic drifts during high-precision isotope-ratio measurements. Optical setup schematic generated using 3DOptix.

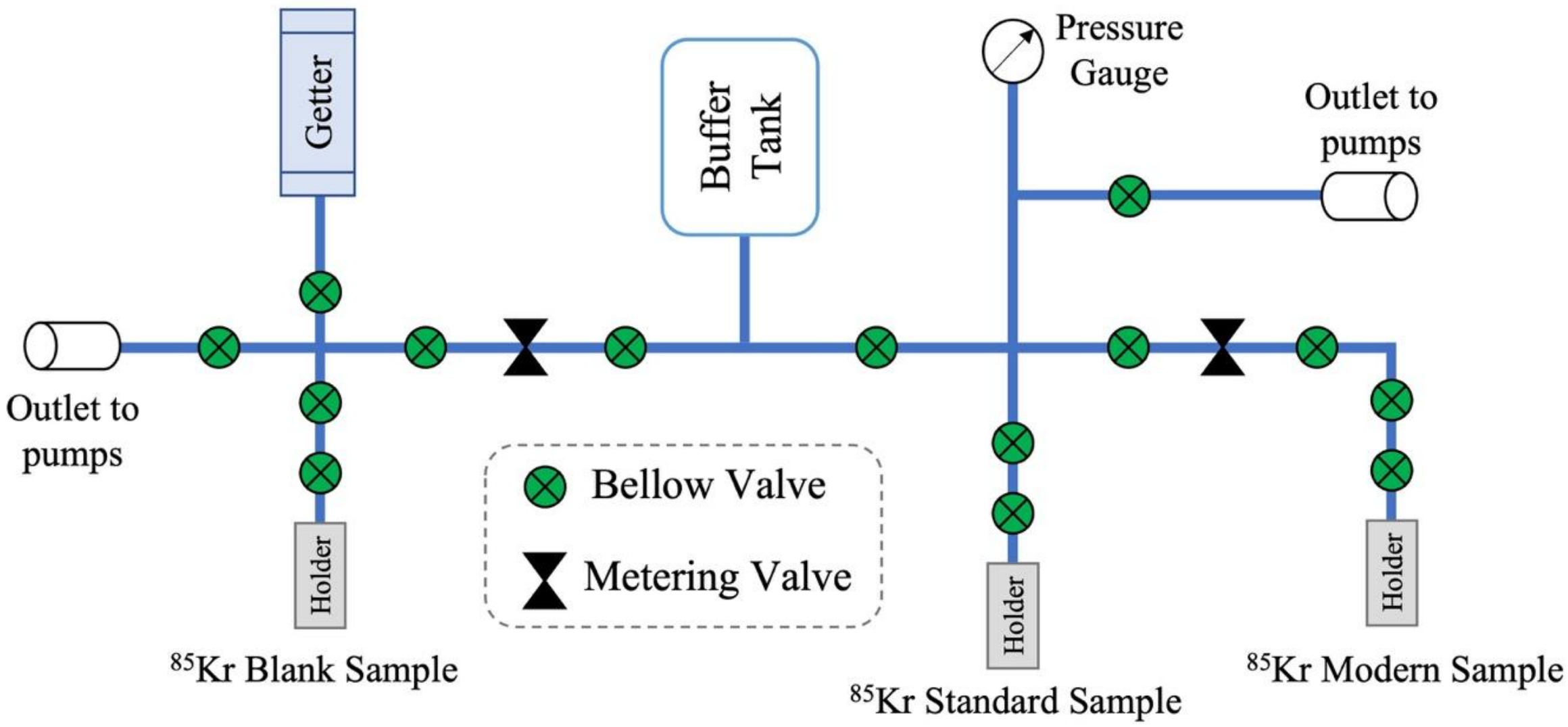

Fig. S3. Schematic diagram of mixing system. A modern krypton sample with known ^{85}Kr activity and ^{85}Kr blank sample are introduced into the mixing manifold through separate holders. The amount of gas transferred from each holder is determined from pressure changes measured with the gauge, allowing standard samples with prescribed ^{85}Kr /Kr ratios to be prepared. The resulting standards are used to evaluate the linearity and precision of the high-precision ATTA system.

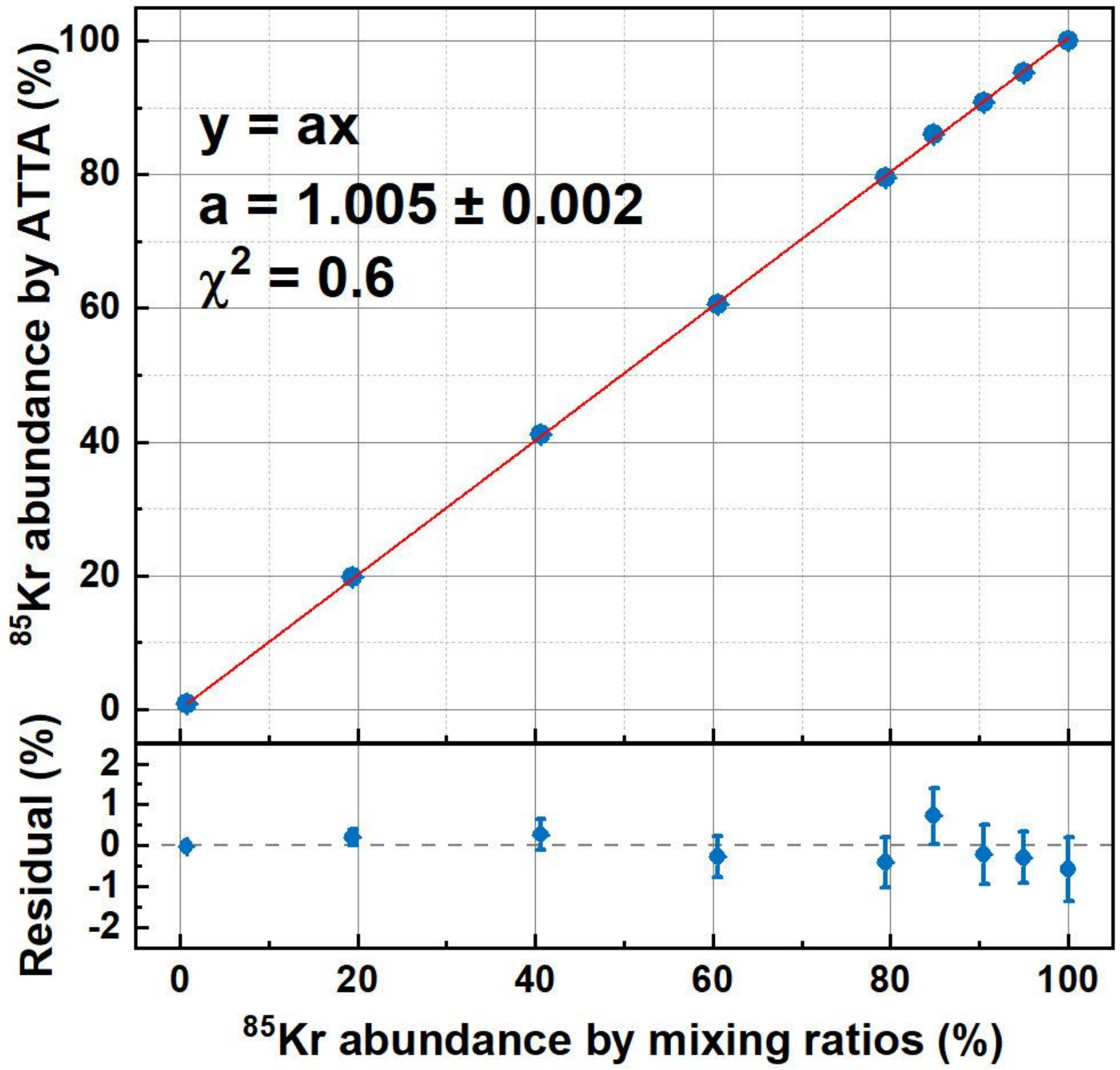

Fig. S4. ^{85}Kr/^{83}Kr ratios of standard samples. Results measured by ATTA are compared with those calculated based on mixing ratios. A proportional function is used to fit the data. The fit residuals are shown in the lower panel in the units of percentage of the modern level. All measurements were made in March 2024.

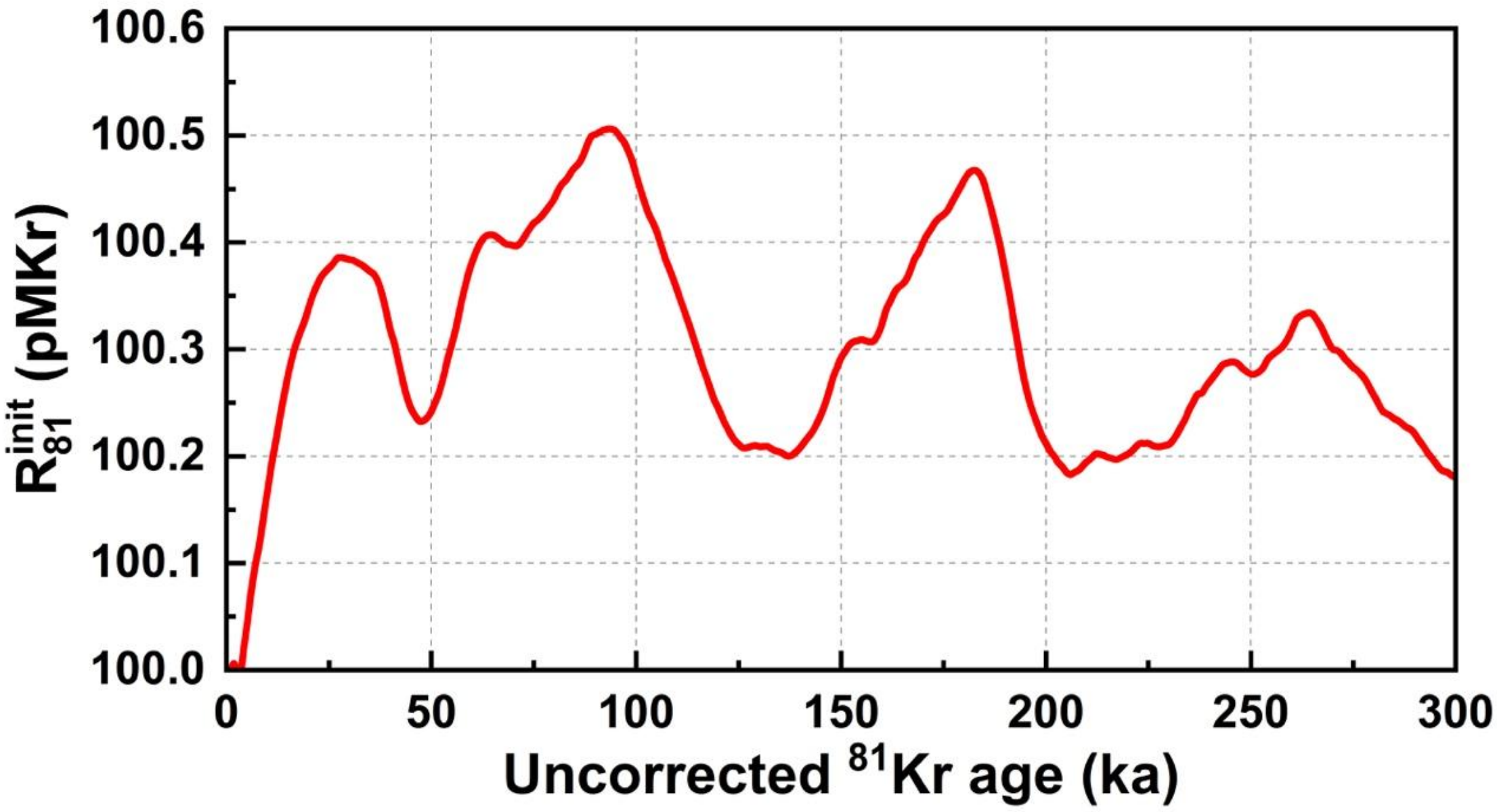

Fig. S5. Atmospheric ^{81}Kr input curve. The curve represents the modeled temporal variation in initial atmospheric ^{81}Kr abundance relative to the modern level. The input function accounts for changes in cosmogenic ^{81}Kr production and is used to correct measured groundwater ^{81}Kr abundances before age calculation. Variations in initial atmospheric ^{81}Kr abundance over the Late Pleistocene are less than 0.5%, resulting in only minor corrections to the calculated ^{81}Kr ages.

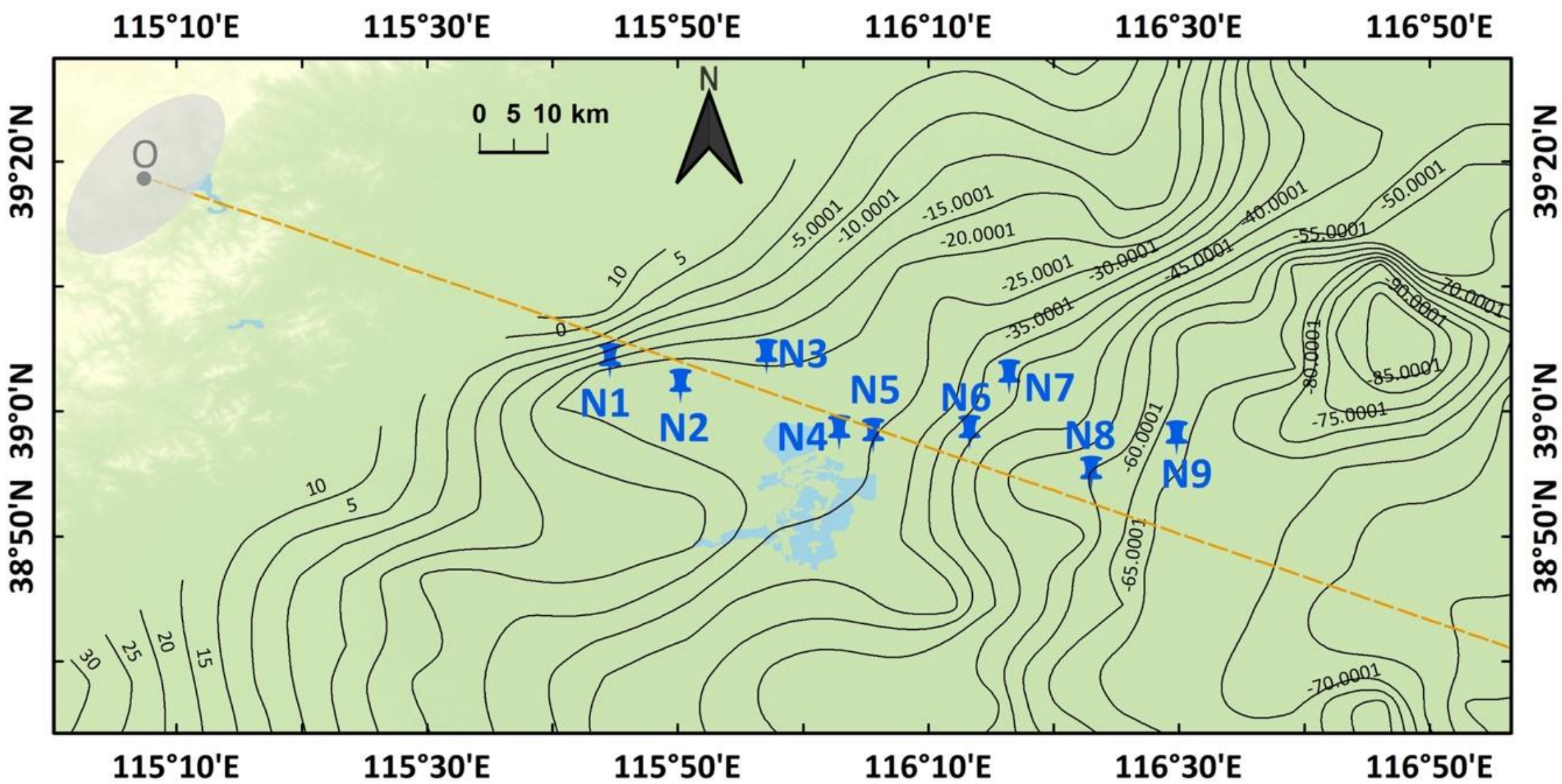

Fig. S6. Recharge area and groundwater-level contour lines. The grey-shaded region is the location of recharge area and point O marks its center. Groundwater-level contours show the spatial distribution of groundwater levels across the study area. The yellow dashed line indicates the location of hydrological profile shown in Fig. 1.

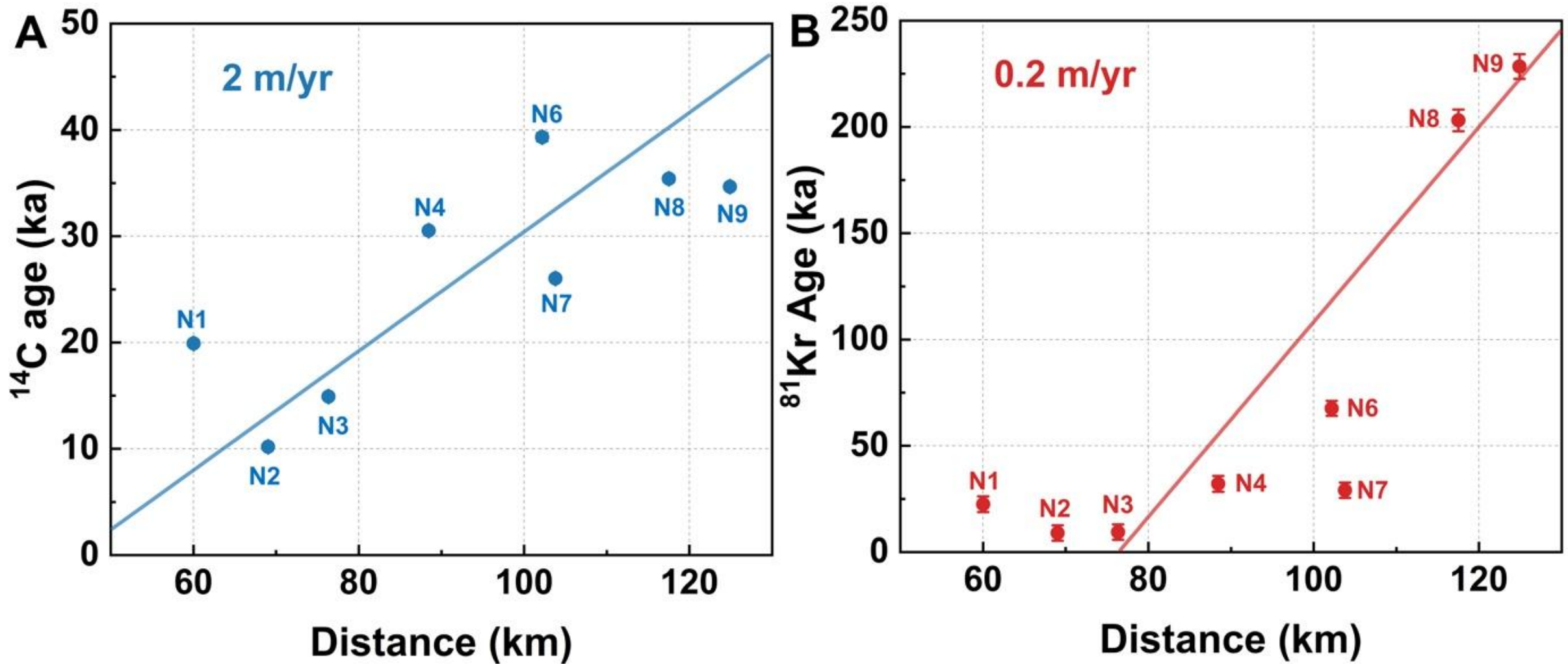

Fig. S7. Apparent groundwater-flow velocity. (**A**) Apparent groundwater-flow velocity based on ^{14}C ages. (**B**) Apparent groundwater-flow velocity based on ^{81}Kr ages. Linear fits to the distance–age relationships yield apparent velocities of approximately 2 m/yr from ^{14}C ages and 0.2 m/yr from ^{81}Kr ages.

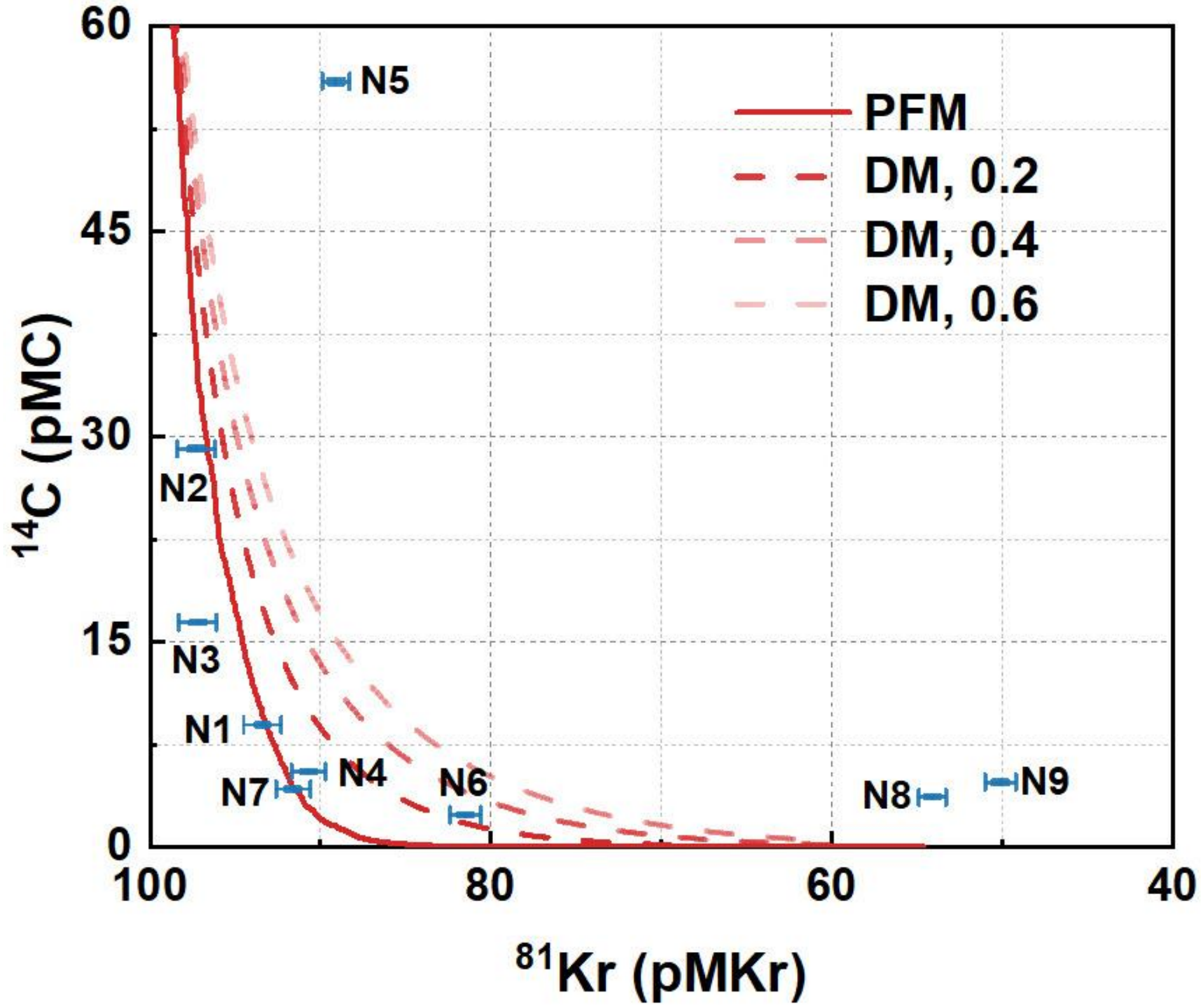

Fig. S8. TracerLPM calculation results. Model calculations compare the expected ^{14}C and ^{81}Kr apparent ages under piston-flow and dispersion conditions.

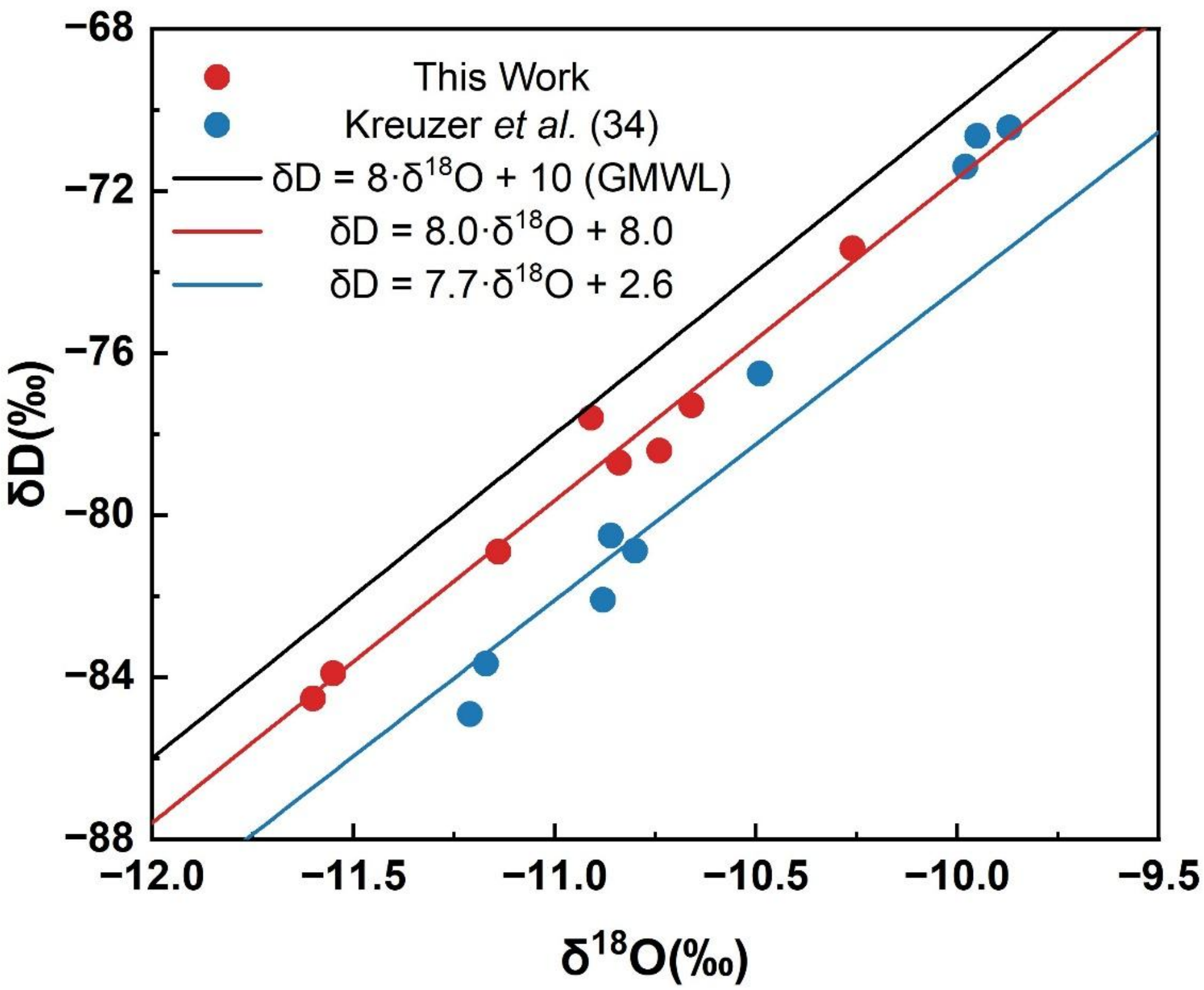

Fig. S9. The relationship between $\delta^{18}O$ and δD in the North China Plain. The red dots represent data collected in this study, and the blue dots are data from Kreuzer *et al.* *(34)*. The black line shows the global meteoric water line (GMWL). The red line is the linear fit to the data from this study and represents the local meteoric water line (LMWL) of this work. The blue line is the LMWL from Kreuzer *et al.* *(34)*.

Table S1. Relative abundance of standard samples.

$No.$	1	2	3	4	5	6	7	8	9
$R_{85}(\%)$	0.72	19.45	40.60	60.52	79.42	84.84	90.51	94.96	100.00
$\delta R_{85}(\%)$	0.05	0.08	0.12	0.12	0.15	0.15	0.17	0.18	0.00

Table S2. Basic information of nine wells and the analysis results of isotopes

Well	$Depth(m)$	pH	T(°C)	$Alkalinity$ $(CaCO3,\ mg/L)$	HCO_3^- (mol/L)	$\delta^{13}C$(‰)	$^{14}C(pMC)$	$R_{85}(\%)$	$R_{81}(pMKr)$	$\delta^{18}O$(‰)	δD (‰)
N1	310	7.74	19.0	226	4.50E-03	-10.35	3.80 ± 0.05	1.2 ± 0.2	93.8 ± 1.0	-10.66	-77.29
N2	300	7.61	18.5	215	4.28E-03	-11.69	13.81 ± 0.08	2.1 ± 0.2	97.5 ± 1.0	-10.26	-73.42
N3	317	8.33	16.8	170	3.34E-03	-13.28	10.16 ± 0.07	0.7 ± 0.2	97.4 ± 1.0	-10.91	-77.60
N4	300	8.46	26.6	168	3.25E-03	-10.93	2.49 ± 0.05	0.7 ± 0.2	91.1 ± 1.0	-11.60	-84.53
N5_1	320	7.86	22.9	238	4.72E-03	-11.95	27.17 ± 0.10	2.5 ± 0.2	89.0 ± 1.0	-10.86	-79.24
N5_2	320	7.86	22.9	238	4.72E-03	-11.95	27.17 ± 0.10	3.5 ± 0.3	90.2 ± 1.1	-10.86	-79.24
N6	320	8.43	18.8	215	4.19E-03	-8.53	0.86 ± 0.04	0.6 ± 0.1	81.9 ± 0.9	-11.14	-80.90
N7	300	8.47	20.0	193	3.75E-03	-9.63	1.71 ± 0.05	0.7 ± 0.2	92.0 ± 1.0	-11.55	-83.90
N8	300	8.47	21.1	251	4.87E-03	-8.96	1.38 ± 0.05	0.6 ± 0.1	54.5 ± 0.8	-10.74	-78.41
N9	306	8.54	17.9	280	5.42E-03	-7.43	1.51 ± 0.05	2.6 ± 0.3	51.5 ± 0.8	-10.84	-78.71

Table S3. Major-ion concentrations of groundwater samples.

$Well$	K^+ (mg/L)	Na^+ (mg/L)	Ca^{2+} (mg/L)	Mg^{2+} (mg/L)	Cl^- (mg/L)	SO_4^{2-} (mg/L)	NO_3^- (mg/L)
$N1$	1.66	53.90	35.50	19.50	12.40	28.50	4.07
$N2$	1.67	46.40	31.50	16.20	8.90	16.20	2.66
$N3$	1.51	54.50	15.30	7.57	3.50	11.10	<0.064
$N4$	0.64	97.70	5.08	2.30	17.70	31.40	6.21
$N5$	0.60	146.00	22.00	11.00	70.90	49.10	19.50
$N6$	0.24	116.00	5.30	1.98	16.00	23.80	<0.064
$N7$	0.26	108.00	6.43	1.90	17.70	27.70	1.15
$N8$	0.33	170.00	4.79	2.11	74.50	23.40	<0.064
$N9$	0.29	170.00	5.35	3.11	51.80	28.40	<0.064

Table S4. Corrections applied to measured ^{81}Kr abundances and corresponding ^{81}Kr ages.

$Well$	$R_{81}(pMKr)$	$R'_{81}(pMKr)$	$R_{81}^{init}\ (pMKr)$	$R_{81}^{corr}(pMKr)$	$^{81}Kr\ age\ (ka)$
$N1$	93.8 ± 1.0	93.8 ± 1.1	100.4	93.4 ± 1.1	$22.5^{+3.7}_{-3.7}$
$N2$	97.5 ± 1.0	97.4 ± 1.1	100.1	97.3 ± 1.1	$9.0^{+3.7}_{-3.6}$
$N3$	97.4 ± 1.0	97.3 ± 1.1	100.1	97.2 ± 1.1	$9.4^{+3.7}_{-3.6}$
$N4$	91.1 ± 1.0	91.1 ± 1.0	100.4	90.7 ± 1.0	$32.1^{+3.7}_{-3.7}$
$N5\_1$	89.0 ± 1.0	88.7 ± 1.1	100.3	88.9 ± 0.8	$38.9^{+3.0}_{-3.0}$
$N5\_2$	90.2 ± 1.1	89.8 ± 1.2	100.4		
$N6$	81.9 ± 0.9	81.8 ± 0.9	100.4	81.5 ± 0.9	$67.6^{+3.5}_{-3.5}$
$N7$	92.0 ± 1.0	91.9 ± 1.0	100.4	91.6 ± 1.0	$29.1^{+3.7}_{-3.6}$
$N8$	54.5 ± 0.8	54.2 ± 0.8	100.2	54.1 ± 0.8	$203.1^{+5.2}_{-5.1}$
$N9$	51.5 ± 0.8	50.2 ± 0.9	100.2	50.1 ± 0.9	$228.4^{+5.9}_{-5.8}$

Table S5. PHREEQC calculation results.

$Well$	$TDIC$ (mol/L)	HCO_3^- (mol/L)	CO_2 (mol/L)	CO_3^{2-} (mol/L)
$N1$	$4.67E-03$	$4.40E-03$	$1.29E-05$	$1.80E-04$
$N2$	$4.52E-03$	$4.21E-03$	$8.90E-06$	$2.35E-04$
$N3$	$3.38E-03$	$3.28E-03$	$3.37E-05$	$3.63E-05$
$N4$	$3.31E-03$	$3.21E-03$	$5.61E-05$	$2.27E-05$
$N5$	$4.86E-03$	$4.64E-03$	$2.02E-05$	$1.34E-04$
$N6$	$4.26E-03$	$4.14E-03$	$5.77E-05$	$3.49E-05$
$N7$	$3.81E-03$	$3.70E-03$	$5.79E-05$	$2.80E-05$
$N8$	$4.96E-03$	$4.80E-03$	$8.03E-05$	$3.51E-05$
$N9$	$5.51E-03$	$5.33E-03$	$9.75E-05$	$3.49E-05$

Table S6. Corrected ^{14}C content using Han-Plummer method.

$Well$	$R_{14}\ (pMC)$	$R_{14}^{init}(pMC)$	$^{14}C\ Model$	$R_{14}^{corr}(pMC)$	$^{14}C\ age\ (ka)$
$N1$	$3.80\ \pm\ 0.05$	42.27	$Eichinger$	8.99 ± 0.12	$19.91^{+0.11}_{-0.11}$
$N2$	$13.81\ \pm\ 0.08$	47.39	$Eichinger$	29.14 ± 0.17	$10.19^{+0.05}_{-0.05}$
$N3$	$10.16\ \pm\ 0.07$	61.68	$Mook$	16.47 ± 0.11	$14.91^{+0.06}_{-0.06}$
$N4$	$2.49\ \pm\ 0.05$	44.68	$Eichinger$	5.57 ± 0.11	$23.87^{+0.17}_{-0.16}$
$N5$	$27.17\ \pm\ 0.10$	48.56	$Eichinger$	55.95 ± 0.21	$4.80^{+0.03}_{-0.03}$
$N6$	$0.86\ \pm\ 0.04$	35.62	$Eichinger$	2.41 ± 0.11	$30.78^{+0.39}_{-0.38}$
$N7$	$1.71\ \pm\ 0.05$	39.79	$Eichinger$	4.30 ± 0.13	$26.02^{+0.25}_{-0.24}$
$N8$	$1.38\ \pm\ 0.05$	37.18	$Eichinger$	3.71 ± 0.13	$27.23^{+0.31}_{-0.29}$
$N9$	$1.51\ \pm\ 0.05$	31.52	$Eichinger$	4.79 ± 0.16	$25.12^{+0.28}_{-0.27}$

Table S7. Initial ^{14}C content estimated using different correction models.

$Well$	$Tamers$ (pMC)	$Pearson$ (pMC)	$Mook$ (pMC)	$Fontes\ and\ Garnier$ (pMC)	$Eichinger$ (pMC)
$N1$	52.06	44.72	22.08	44.53	42.27
$N2$	52.76	49.77	36.10	49.70	47.39
$N3$	50.57	55.77	61.68	58.51	53.70
$N4$	50.37	46.91	36.75	46.77	44.68
$N5$	51.49	50.75	43.46	50.73	48.56
$N6$	50.44	37.85	4.83	37.54	35.62
$N7$	50.39	42.00	19.22	41.77	39.79
$N8$	50.38	39.47	12.53	39.15	37.18
$N9$	50.34	33.70	−9.26	33.32	31.52

Table S8. Corrected ^{14}C contents calculated using different models.

$Well$	$Tamers$ (pMC)	$Pearson$ (pMC)	$Mook$ (pMC)	$Fontes\ and\ Garnier$ (pMC)	$Eichinger$ (pMC)
$N1$	7.30 ± 0.10	8.50 ± 0.11	17.21 ± 0.23	8.53 ± 0.11	8.99 ± 0.12
$N2$	26.18 ± 0.15	27.75 ± 0.16	38.25 ± 0.22	27.79 ± 0.16	29.14 ± 0.17
$N3$	20.09 ± 0.14	18.22 ± 0.13	16.47 ± 0.11	17.36 ± 0.12	18.92 ± 0.13
$N4$	4.94 ± 0.10	5.31 ± 0.11	6.78 ± 0.14	5.32 ± 0.11	5.57 ± 0.11
$N5$	52.77 ± 0.19	53.54 ± 0.20	62.52 ± 0.23	53.56 ± 0.20	55.95 ± 0.21
$N6$	1.70 ± 0.08	2.27 ± 0.11	17.81 ± 0.83	2.29 ± 0.11	2.41 ± 0.11
$N7$	3.39 ± 0.10	4.07 ± 0.12	8.90 ± 0.26	4.09 ± 0.12	4.30 ± 0.13
$N8$	2.74 ± 0.10	3.50 ± 0.13	11.01 ± 0.40	3.52 ± 0.13	3.71 ± 0.13
$N9$	3.00 ± 0.10	4.48 ± 0.15	-16.31 ± 0.54	4.53 ± 0.15	4.79 ± 0.16
